# T cell expressions of aberrant gene signatures and Co-inhibitory receptors (Co-IRs) as predictors of renal damage and lupus disease activity

**DOI:** 10.1186/s12929-024-01024-7

**Published:** 2024-04-22

**Authors:** Chin-Man Wang, Yeong-Jian Jan Wu, Jian-Wen Zheng, Li Yu Huang, Keng Poo Tan, Ji-Yih Chen

**Affiliations:** 1 Department of Rehabilitation, Chang Gung Memorial Hospital, Chang Gung University College of Medicine, No. 5, Fu‐Shin St. Kwei‐Shan, Taoyuan, Republic of China; 2 Department of Medicine, Division of Allergy, Immunology and Rheumatology, Chang Gung Memorial Hospital, Chang Gung University College of Medicine, Taoyuan, Taiwan No. 5, Fu-Shin St. Kwei-Shan, Republic of China

**Keywords:** SLE, Transcriptome, Co-inhibitory receptors (Co-IR)

## Abstract

**Background:**

Systemic lupus erythematosus (SLE) is distinguished by an extensive range of clinical heterogeneity with unpredictable disease flares and organ damage. This research investigates the potential of aberrant signatures on T cell genes, soluble Co-IRs/ligands, and Co-IRs expression on T cells as biomarkers for lupus disease parameters.

**Methods:**

Comparative transcriptome profiling analysis of non-renal and end-stage renal disease (ESRD) phenotypes of SLE was performed using CD4 + and CD8 + cDNA microarrays of sorted T cells. Comparing the expression of Co-IRs on T cells and serum soluble mediators among healthy and SLE phenotypes.

**Results:**

SLE patients with ESRD were downregulated CD38, PLEK, interferon-γ, CX3CR1, FGFBP2, and SLCO4C1 transcripts on CD4 + and CD8 + T cells simultaneously and NKG7, FCRL6, GZMB/H, FcγRIII, ITGAM, Fas ligand, TBX21, LYN, granulysin, CCL4L1, CMKLR1, HLA-DRβ, KIR2DL3, and KLRD1 in CD8 T cells. Pathway enrichment and PPI network analyses revealed that the overwhelming majority of Differentially Expressed Genes (DEGs) have been affiliated with novel cytotoxic, antigen presentation, and chemokine-cell migration signature pathways. CD8 + GZMK + T cells that are varied in nature, including CD161 + Mucosal-associated invariant T (MAIT) cells and CD161- aged-associated T (Taa) cells and CD161-GZMK + GZMB + T cells might account for a higher level of GZMK in CD8 + T cells associated with ESRD. SLE patients have higher TIGIT + , PD1 + , and lower CD127 + cell percentages on CD4 + T cells, higher TIM3 + , TIGIT + , HLA-DR + cell frequency, and lower MFI expression of CD127, CD160 in CD8 T cells. Co-IRs expression in T cells was correlated with soluble PD-1, PDL-2, and TIM3 levels, as well as SLE disease activity, clinical phenotypes, and immune-therapy responses.

**Conclusion:**

The signature of dysfunctional pathways defines a distinct immunity pattern in LN ESRD patients. Expression levels of Co-IRs in peripheral blood T cells and serum levels of soluble PD1/PDL-2/TIM3 can serve as biomarkers for evaluating clinical parameters and therapeutic responses.

**Supplementary Information:**

The online version contains supplementary material available at 10.1186/s12929-024-01024-7.

## Introduction

Systemic lupus erythematosus (SLE) is a chronic, debilitating, autoimmune disorder that is defined by systemic inflammation. The innate and adaptive immune systems substantially contribute to the imbalanced immune reaction to self-antigens, which promotes immune tolerance loss and systemic autoimmunity toward nuclear autoantigens [1]. Recent research emphasizes understanding the biological processes enabling immune cell differentiation, which leads to the identification of novel potential prospects for immune-mediated blockade therapies and aids in the clinical course of SLE [2, 3]. Diverse functional pathways and domains in proteins have been attributed to the organ-specific development of SLE. Emerging important research seeks to identify the precise mechanisms that contribute to the wide variety of clinical disease risks associated with SLE, which impacts multiple organs and ultimately results in a high mortality rate [4]*.* Lupus nephritis (LN) is defined by glomerulonephritis and tubulointerstitial inflammation among individuals diagnosed with SLE within 5 years, and 5–20% of those diagnosed with LN advance to end-stage renal disease (ESRD) [5–7]. LN is a potentially fatal autoimmune disease that necessitates early and accurate diagnosis as well as prompt treatment initiation for improving outcomes [6, 8]. The purposes of therapy are for the individual's long-term survival, the prevention of flares and organ injury, and the improvement of quality of life [9, 10]. Identifying the activity of SLE facilitates monitoring of the disease and the appraisal of therapeutic interventions. The enhanced comprehension of signaling and gene regulation deficiencies will result in the discovery of novel therapeutic targets and predictive biomarkers for LN.

T cells have become increasingly accepted as key contributors to the development of SLE [2, 3, 11, 12]. The functions of varied effector, memory, exhausted and regulatory T cells are controlled by distinct pathways [13]. With the goal to improve the treatment and outcome of this complicated disease, it is necessary to identify the molecular and genetic defects of malfunctioning signaling pathways that lead to dysfunctional SLE T cells [2]**.** It is feasible to speculate that diverse aberrant gene expression and specific immune functional changes in T cells result in disruption of immune tolerance and, ultimately, autoimmune responses in SLE [14]. This study aimed to investigate novel immune-regulatory pathways that predict the heterogeneity and activity of lupus disease based on gene aberrant signatures and dysfunctions of Co-inhibitory receptors (Co-IRs) on T cells.

## Materials and methods

### Description of the characteristics of study populations and cohorts

Patients for this study were recruited primarily from Chang Gung Memorial Hospital's Rheumatology Clinics. Rheumatologists who confirmed SLE in all examined patients based on American College of Rheumatology criteria [15, 16]. Specific phenotypes of SLE were correlated with biological data. Ninety one patients with SLE (age, 23–80 years; 49.9 ± 11.7 median years) and 27 Health Controls (HCs) (age, 27–52 years; 36.3 ± 5.7 years) were enrolled. There were 68 patients with SLE without nephritis and 23 patients with nephritis; 25 had SLEDAI ≥ 6, compared to 66 < 6. Regarding the immunologic assays, this cohort observed Anti-dsDNA antibody: 35 ≧130 vs. 55 < 130; C3: 69 ≧70 vs. 22 < 70; C4: 74 ≧10 vs. 17 < 10; Anti-RNP antibody: 28 positive vs. 52 negative; Anti-SM antibody, 16 positive vs. 64 negative; Anti-SSA antibody, 51 positive vs. 31 negative; Anti-SSB antibody, 15 positive vs. 67 negative; Anti-ACA IgG antibody, 10 positive vs. 61 negative; Anti-ACA IgM antibody, 4 positive vs. 61 negative. Steroid (≥ 10 mg 67, 10-20 mg 23, > 20 mg 11), azathioprine (≥ 50 mg 13; > 50 mg 4), hydroxychloroquine (≥ 200 mg 35; > 200 mg 23), and mycophenolate (≤ 1000 mg 5; ≥ 1000 mg 8) are utilized as immune modulating drugs (Supplementary Table 1). Fourteen patients with SLE underwent a follow-up immune evaluation, with a median follow-up of 12.5 months after SLE diagnosis (range, 7–23; 13.0** ± **4.2 months).

### Isolation and enrichment of CD4 and CD8 T cells

Peripheral blood mononuclear cells (PBMCs) from SLE patients were isolated using Ficoll-Hypaque gradients (GE Healthcare, Uppsala, Sweden) in accordance to the manufacturer's protocol. PBMCs were separated by flow cytometry toward CD4 + T cells and CD8 + T cells.

### RNA isolation

The total RNA of mononuclear cells was isolated using the TRIzol total RNA isolation reagent. With the Superscript pre-amplification system VILO cDNA Synthesis reagent (Invitrogen Life Technologies), 5ug of total MNC RNA was used to synthesize complementary DNA (cDNA).

### The cDNA microarray analysis

The CD4 + and CD8 + T-cell specimens that satisfied the criteria for RNA quality were sent for microarray analysis. The Bioanalyzer 2100 (Agilent Technologies, Santa Clara, California, United States) was used to evaluate the quantity and purity of RNA. Chang Gung Memorial Hospital (CGMH) utilized a GeneChip Human Genome U133 Plus2 array (Affymetrix, Santa Clara, California, United States) for microarray analysis. RNA was separated and incorporated into sequencing libraries with the TruSeq Stranded Total RNA Sample Preparation Kit KAPA mRNA HyperPrep Kit and KAPA Dual-Indexed Adapter Kit (Illumina, San Diego, California, United States) with ribosomal depletion using Ribo-Zero, and then analyzed on an Illumina NovaSeq 6000. On average, 50 million readings were generated per sample. Using FastQC Prefiltering, the integrity of raw reads was analyzed.

### Functional annotation and pathway enrichment analysis of Differential Expression Gene (DEG)

The R package DESeq2 was used to adjust raw expression counts for library size (version 1.16.1). A pre-filtering of low-count genes was conducted to retain only genes with at least 50 total reads. Outliers were identified with the help of principal component analysis (PCA). The first five principal components (PCs) of each sample were extracted and correlated with clinical and technical information. Supervised hierarchical clustering of the SLE cohort utilizing subgroup-defining genes (> twofold statistically significant differential expression, *p* < 0.05, FDR < 0.05). The subsequent analysis was conducted using the Database for Annotation, Visualization and Integrated Discovery (DAVID; http://david.abcc.ncifcrf.gov/), Kyoto Encyclopedia of Gene and Genomes (KEGG; http://www.genome.jp/kegg) Bioinformatics databases as well as the UCSC genome browser tool. PPI (protein–protein interaction) network analysis was performed to identify the immune response gene modules of CD4 + and CD8 + T cells that contribute to LN pathogenesis based on the STRING v11.5 database (STRING, https://www.string-db/org). After data normalization and quality control, CD8 T cells prominent DEGs were identified between SLE patients with ESRD and those without nephritis. KEGG biopathways and Gene Ontology (GO) utilized all identified 52 CD8 T cells prominent DEGs to conduct pathway enrichment analyses for the specific biological functions of molecular function (MF), cellular component (CC), and biological process (BP). The GO terms and pathways were deemed significantly enriched when the FDR-adjusted P value was less than 0.05.

### Flow cytometry measurement of T cell phenotype surface marker

Using multicolor calibration particles (BD Biosciences) in conjunction with saturated amounts of the following antibodies: CD279 (FITC Mouse Anti-Human CD279 Clone MIH4); Tim3 (PE Mouse Anti-Human TIM-3 (CD366) Clone 7D3); CD4 (PE-Cy™5 Mouse Anti-Human CD4 Clone RPA-T40; CD8 (APC Mouse Anti-Human CD8 Clone RPA-T8); CTLA-4 (Human CTLA-4 Alexa Fluor® 488-conjugated Antibody Clone # 2188A); LAG-3 (PE Mouse Anti-Human LAG-3 (CD223) Clone T47-530); CD127 (FITC Mouse Anti-Human CD127 Clone HIL-7R-M21); TIGIT (PE anti-human TIGIT (VSTM3) Antibody Clone A15153G); CD160 (Alexa Fluor® 488 Mouse Anti-Human CD160 Clone BY55); CD244 (PE Mouse Anti-Human CD244 Clone 2–69); HLA-DR (FITC Mouse Anti-Human HLA-DR Clone G46-6); CD38 (PE Mouse Anti-Human CD38 Clone HIT2); CD3 (PE-Cy™5 Mouse Anti-Human CD3 Clone HIT3a); CD103 (Brilliant Violet 421™ anti-human CD103 (Integrin αE) Clone Ber-ACT8); CD161(BD Horizon™ BV510 Mouse Anti-Human CD161 Clone DX12); Granzyme B (BD Pharmingen™ PE Mouse Anti-Human Granzyme B Clone GB11); Granzyme K (APC anti-human Granzyme K Antibody Clone GM26E7), CD197(CCR7) (BD Pharmingen™ PE-Cy™7 Rat Anti-Human CCR7 (CD197) Clone 3D12) and CD45RA (BD Horizon™ BV711 Mouse Anti-Human CD45RA Clone HI100), reactions were standardized. Following washing, the pellets were resuspended in cold staining buffer and analyzed using LSRFortessa and FACSCanto II flow cytometers (BD Biosciences). Using the software from FlowJo, LLC (Tree Star, Ashland, OR, USA) to collect and analyze the cells. The phenotype of immune cell subsets was determined using the HIP protocol of four color flow cytometric analysis.

### Enzyme-linked immunosorbent assay (ELISA) for soluble mediators analysis

ELISA kits (R&D Systems) were used in accordance with the manufacturer's instructions to measure the serum/plasma levels of PD-1, PDL-2 and TIM3.

### ***Human PBMC culture and ***in vitro*** IFN-***${\varvec{\beta}}$*** stimulation***

Ficoll-Paque Plus (Cytiva, Uppsala, Sweden) was utilized to generate PBMCs from whole blood of SLE-LN- and SLE-LN + patients. PBMCs were subsequently cultured in complete RPMI 1640 medium, which was supplemented with 10% FBS and 1% penicillin–streptomycin solution. PBMCs were inoculated into 6-well plates on day two, with 2.0 × 10^6^ cells per well. The cells were then subjected to treatment with recombinant human IFN-β (200 ng/mL; R&D System), either with or without Tofacitinib (200 nM; Pfizer), or were left untreated. Following 120 h of IFN- stimulation, collected cells were subjected to co-IR antibody staining in preparation for flow cytometry analysis. For phosflow, cells were analyzed in the CD3 + lymphocyte population after being stained with p-STAT1 and total STAT1 antibodies according the manufacturer's instructions (BioLegend).

### Statistical analysis

Using Graph Pad Prism 8.4.1, the statistical analyses were conducted. The data was presented as mean plus/minus standard deviation (S.D.) or as a percentage (%). The t-test was used to compare the differences between groups. Using Pearson's correlation coefficient, a correlation analysis was performed. The unpaired t-test was utilized to determine whether there were statistically significant differences between baseline data and those measured before and after therapy. Every aspect stated *P*-values were two-sided and not multiple testing adjusted. The degree of significance was set at *P* < 0.05.

## Results

### *Profiling analysis revealed aberrant CD4* + *and CD8* + *T cell mRNA transcription in SLE phenotypes*

**Fig. 1:**
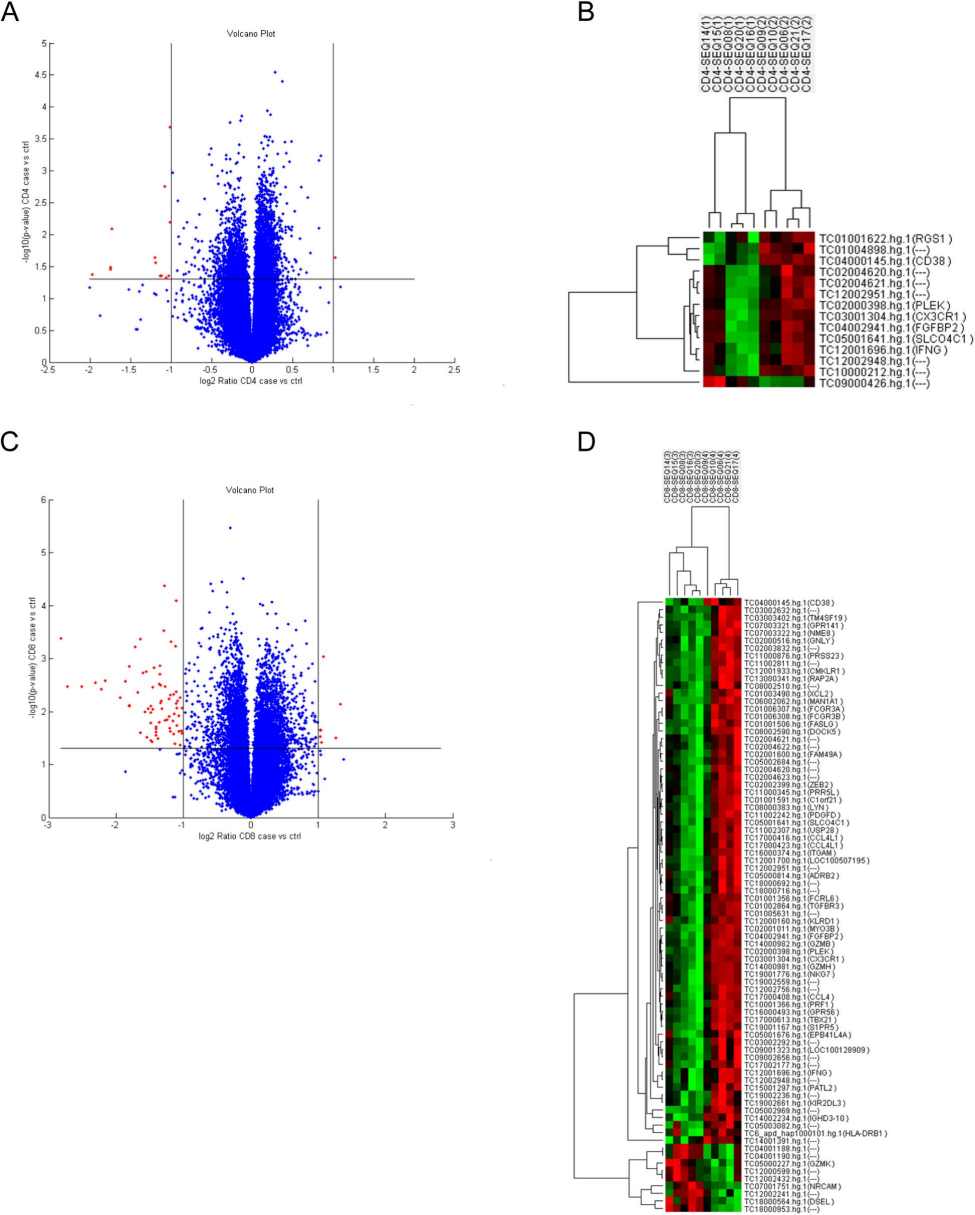
Differential expressed genes (DEGs) in SLE patients with ESRD (14, 15, 08, 20,16) and inactive SLE controls (09, 16, 06, 21, 17) (**A**) Volcano of the DEGs of CD4 T cells. **B** Heatmap of the CD4 T cells DEGs in SLE patients with ESRD and inactive SLE controls. **C** Volcano of the DEGs of CD8 T cells. **D** Heatmap of the 52 DEGs in SLE patients with ESRD and inactive SLE controls

**Fig. 2:**
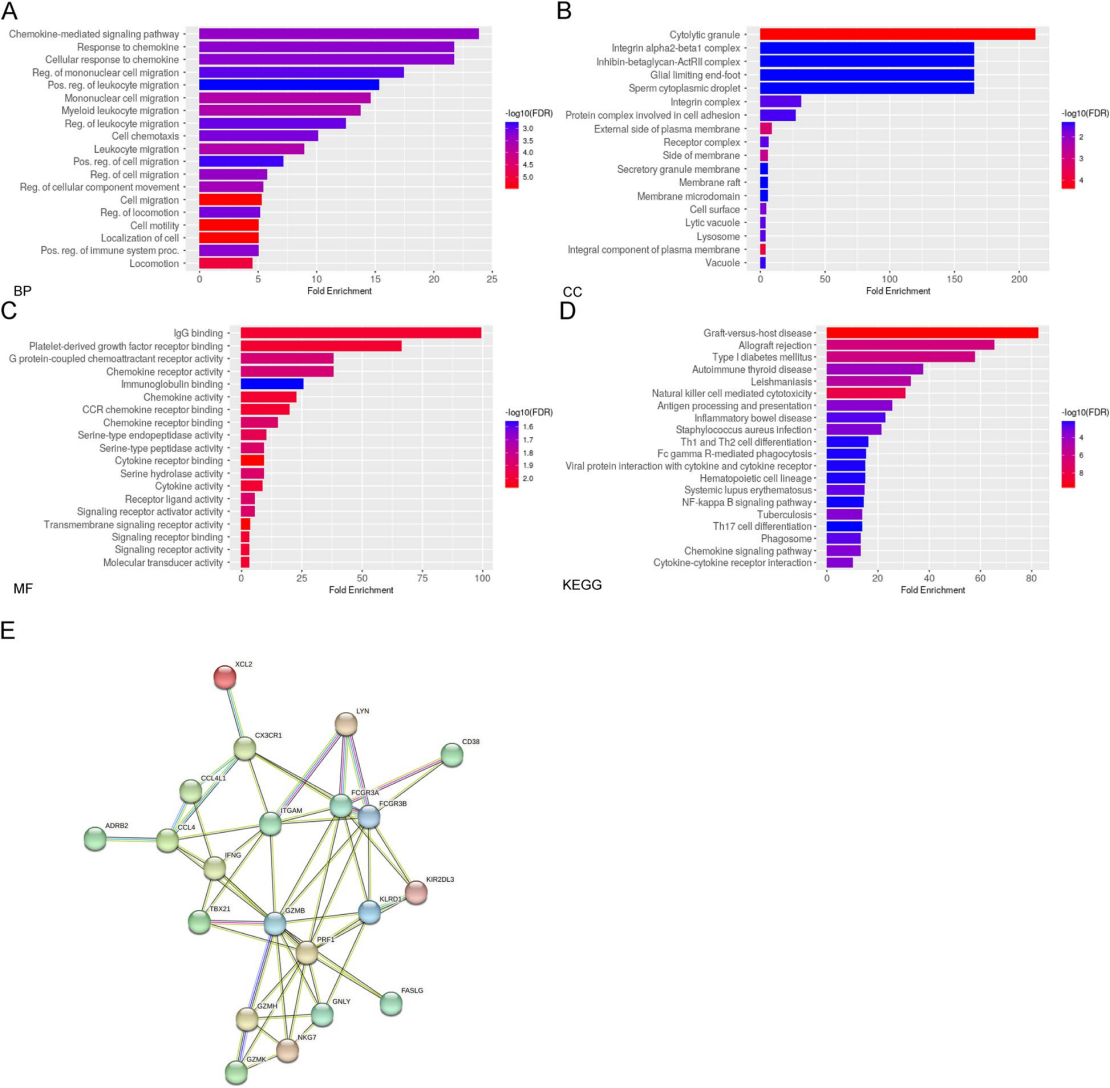
Pathway enrichment analysis of CD8 T cells based on in-depth analyses of our DEGs (**A**) Chemokine mediated signal pathway and cell migration on biological process (BP) (**B**) Cytolytic granule on cellular component (CC) (**C**) Ig binding, platelet derived growth factor receptor binding and chemokine activity on molecular function (MF) (**D**) graft versus host disease on KEGG (**E**) The protein–protein interaction was obtained from the STRING database showed cytotoxicity signature, antigen presentation and chemokine-cytokine interaction influence the pathogenesis of advanced LN

Ten sorted CD4 + T cell and CD8 + T cell specimens from five SLE patients with ESRD (two for 4 years and three for 12 years more) and five patients without LN in the disease course were sent for microarray transcriptomes analysis. The expression levels on CD4 and CD8 T cells were used to execute a hierarchical clustering analysis of T cells. The Volcano plots and hierarchical clustering heat map revealed that LN with ESRD and SLE without LN phenotypes clustered significantly different gene expression levels. The volcano plot and heatmap of the most altered genes, as depicted in Fig. 1, several critical unappreciated heterogeneity immune response genes that revealed a distinctive gene signature profile related to T cells differentiation, activation, and cytotoxicity on CD4 and CD8 T cells. In SLE with ESRD patients, CD38, PLEK (platelet and leukocyte C kinase substrate and the KSTR sequence of amino acids), interferon-γ, CX3CR1, fibroblast growth factor binding protein type 2 (FGFBP2), and solute carrier organic anion transporter family member 4C1 (SLCO4C1) were significantly downregulated on CD4 and CD8 T cells simultaneously. Fc receptor-like 6 (FCRL6), granzyme B (GZMB), GZMH, FcγR3A/3B, integrin subunit alpha M (ITGAM), FAS ligand, TBX21 (T-box transcription factors; also known as T-bet), LYN (Lck/Yes Novel tyrosine kinase, Src kinase family), granulysin, C–C motif chemokine ligand 4 like 1 (CCL4L1), chemerin chemokine-like receptor 1 (CMKLR1), HLA-DRβ1, KIR2DL3 and killer cell lectin like receptor D1 (CD94) transcripts were downregulated, whereas GZMK, NRCAM (Neuronal cell adhesion molecule) and *DSEL* (dermatan sulfate epimerase like) were upregulated in CD8 T cells. Pathway enrichment analysis of CD8 T cells revealed that Chemokine and cell migration were critical on BP (Fig. 2A), cytolytic granule on CC (Fig. 2B), Ig binding and platelet derived growth factor receptor binding on MP (Fig. 2C) and graft versus host disease on KEGG were associated with the majority of genes and fold changes (Fig. 2D). PPI network analysis uncovered the functional interdependence of these crucial pathways (Fig. 2E). As depicted in the Supplemental Figures, the gene list best defining the pathways suggests that the NK cytotoxicity signature (KIR2DL3, CD94, FcγR, perforin, granzyme and Fas/Fas ligand induced apoptosis), graft versus host disease (MHC class II antigen processing and type I interferon and host target tissue injury), and chemokine-cytokine interaction (CCL4 and CX3CR1) significant influence the pathogenesis of advanced LN. Collectively, the aforementioned molecules are responsible for the phenomenon of inflammatory senescence commonly observed in repeatedly activated T cells, resulting in a substantial reduction of CD8 T cell cytotoxicity in LN patients with ESRD.

### *GZMB and GZMK expressing CD8* + *T cells impact on SLE and clinical outcome*

In order to gain greater comprehension of GZMK expression in SLE, we compared GZMK-expressing CD8 + T cells from normal controls, SLE with and without LN. As shown in Table 1, there was no significant difference observed in the percentage and MFI expression of CD45RA-CD197-CD8 + GZMK + T cells between SLE patients and normal controls (20.32 ± 1.872%, (*N* = 38) vs. 19.96 ± 1.685% (*N* = 25), *p* = 0.895; MFI: 3546 ± 273.9 (*N* = 38) vs. 3526 ± 192 (*N* = 25), *p* = 0.9589). Yet, compared to normal controls, the proportion of CD45RA- CD103-CD8 + KLRB(CD161) + GZMK + Mucosal-associated invariant T (MAIT) cells decreased in SLE patients (6.200 ± 1.103%, (*N* = 25) vs. 1.158 ± 0.2279%, (*N* = 38), *p* =  < 0.0001; MFI: 3709 ± 197.6 (*N* = 38) vs. 4323 ± 329.3 (*N* = 25), *p* = 0.9589). The observation of a notable decrease in the quantity of CD8 + CD161 + GZMK + MAIT cells indicates that a segment of these cells may have escaped the bloodstream by migrating to inflamed tissues.

**Table 1:** Expression distribution of CD161 and GZMK on CD8 T cells in SLE, LN + , LN- and normal

Markers	Mean ± SD (Number)				*p* Value			
	Normal	SLE	LN-	LN +	N vs. SLE	N vs. LN-	N vs. LN +	LN- vs. LN +
45RA-197-CD8 + GZMK +	19.24 ± 1.594 (25)	19.18 ± 1.834 (38)	20.92 ± 2.423 (25)	15.85 ± 2.514 (13)	0.983	0.5652	0.2424	0.1933
GZMK MFI in 45RA-197-CD8 + GZMK +	3526 ± 192.0 (25)	3546 ± 273.9 (38)	3841 ± 387.2 (25)	2978 ± 242.4 (13)	0.9589	0.4702	0.0937	0.1368
45RA-CD8 + 103–161 + GZM K + in CD8	6.080 ± 1.100 (25)	1.105 ± 0.2160 (38)	1.280 ± 0.2800 (25)	0.7692 ± 0.3233 (13)	< 0.0001	0.0001	0.0016	0.2676
GZMK MFI in 45RA-CD8 + 103–161 + GZM K +	3709 ± 197.6 (25)	4323 ± 329.3 (38)	4605 ± 376.5 (25)	3781 ± 628.3 (13)	0.1652	0.0403	0.8919	0.2401

**Fig. 3:**
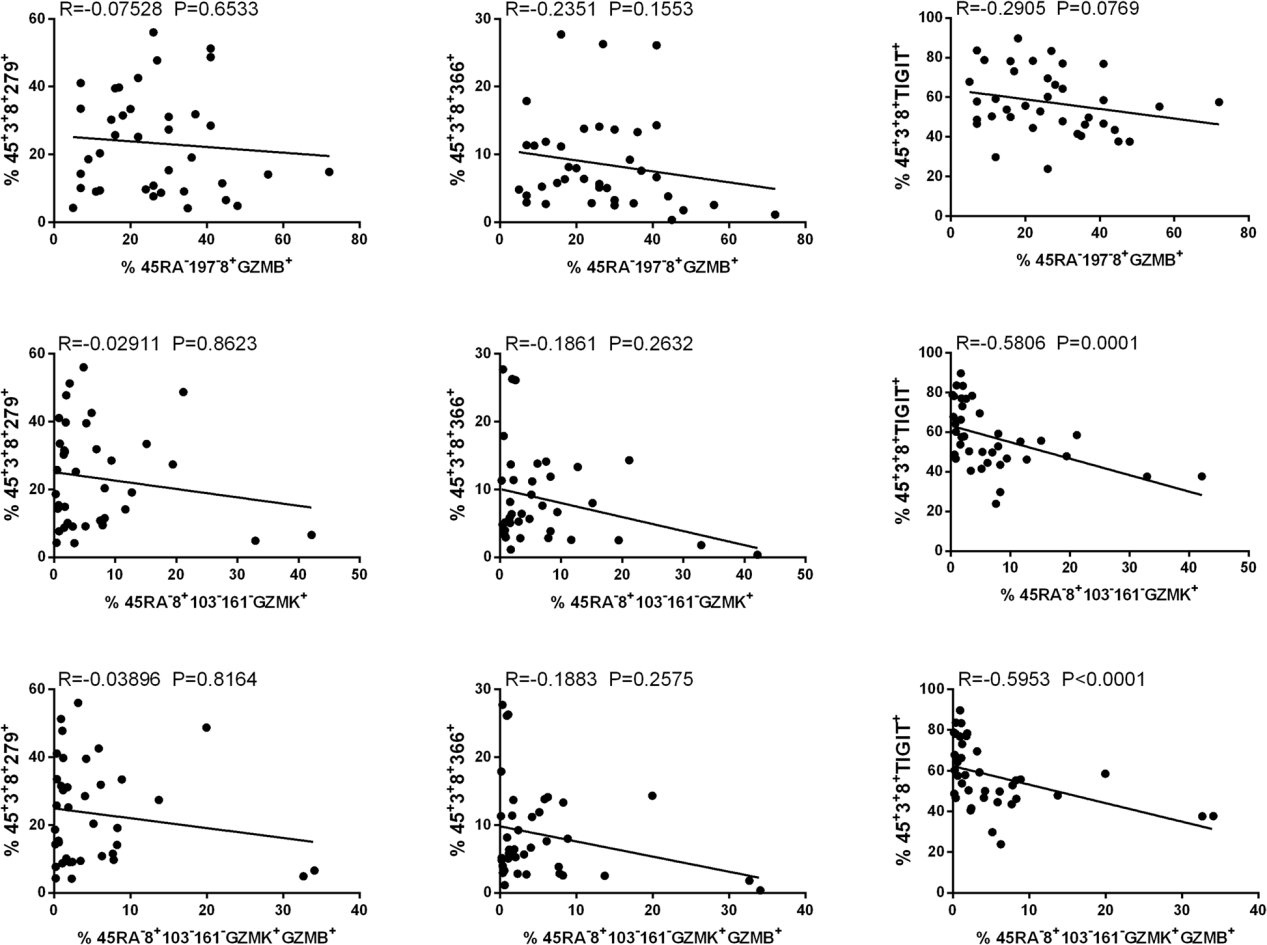
The performance of Co-IRs expression on CD8 T cell correlation with varied CD8 + T cell expressing GZMK and GZMB. The *p* values are represented as follows: *P < 0.05, **P < 0.01, ***P < 0.001, ****P < 0.0001, NS no significance (*P* ≥ 0.05)

To determine the impact of GZMB and GZMK-expressing CD8 + T cells on ESRD, the expression levels of GZMB and GZMK on CD8 T cells from LN with ESRD, healthy controls, and SLE were compared. Table 2 presented that SLE patients possess a surge in CD45RA-CD197-8 + GZMB + CD8 T cells (9.320 ± 1.169%, (*N* = 25) vs. 26.32 ± 2.474%, (*N* = 38), *p* =  < 0.0001), CD45RA-CD8 + CD103-CD161- GZMK + T cells (4.207 ± 0.6274%, (*N* = 25) vs. 6.989 ± 1.461%, (*N* = 38), *p* = 0.1432), and CD45RA-CD8 + CD103-CD161- GZMK + GZMB + T cells (1.874 ± 0.2710%, (*N* = 25) vs. 6.989 ± 1.461%, (*N* = 38), *p* = 0.0365) than healthy controls. It is noteworthy that ESRD patients exhibited greatly greater of CD45RA-CD8 + CD103-CD161- GZMK + T cells (1.874 ± 0.2710%, (*N* = 25) vs. 11.29 ± 4.934%, (*N* = 5), *p* = 0.0082), and CD45RA-CD8 + CD103-CD161- GZMK + GZMB + T cell (1.874 ± 0.2710%, (*N* = 25) vs. 7.846 ± 4.233%, (*N* = 5), *p* = 0.0033) were detected. Additionally, ESRD patients have a slightly higher quantity of CD45RA-CD8 + CD103-CD161 + GZMK + T MAIT cells than other SLE patients (2.070 ± 1.18 (*N* = 5) vs. 1.234 ± 0.2120 (*N* = 38)). Therefore, a diversity of GZMK + CD8 T cells may be responsible for an upsurge in GZMK expression in ESRD patients comparing to other SLE patients. Following this, the expression of Co-IRs (TIGIT, PD-1, and TIM3) on CD8 + cells was correlated with that of CD8 + cells expressing GZMK and GZMB. Figure 3 showed that there was a moderate negative correlation between the expressions of CD8 + TIGIT + T cells with CD45RA-CD8 + CD103-CD161- GZMK + T cells (*r* = -0.5806; *p* = 0.0001) and CD45RA- CD8 + CD103-CD161-GZMK + GZMB + CD8 T cells (*r* = -0.5953; *p* =  < 0.0001). Nevertheless, additional longitudinal research is necessary in order to ascertain the serial long-term effects.

**Table 2:** Expression distribution of CD8 T cells for CD161, GZMB, and GZMK in SLE, ESRD and normal

Markers	Mean ± SD (Number)		*P* Value	Mean ± SD (Number)		*P* Value
	Normal	ESRD-SLE		Normal	SLE	
45RA-197–8 + GZMB + in CD8	9.320 ± 1.169 (25)	19.20 ± 4.748 (5)	0.0057	9.320 ± 1.169 (25)	26.32 ± 2.474 (38)	< 0.0001
45RA-197–8 + GZMK + in CD8	19.96 ± 1.685 (25)	20.20 ± 5.257 (5)	0.9569	19.96 ± 1.685 (25)	19.18 ± 1.834 (38)	0.7702
45RA-8 + 103–161-GZMK + in CD8	4.207 ± 0.6274 (25)	11.29 ± 4.934 (5)	0.0082	4.207 ± 0.6274 (25)	6.989 ± 1.461 (38)	0.1432
45RA-8 + 103–161-GZMK + GZMB + in CD8	1.874 ± 0.2710 (25)	7.846 ± 4.233 (5)	0.0033	1.874 ± 0.2710 (25)	5.295 ± 1.282 (38)	0.0365
45RA-8 + 103–161 + GZMK + in CD8	6.067 ± 1.066 (25)	2.070 ± 1.182 (5)	0.1164	6.067 ± 1.066 (25)	1.234 ± 0.2120 (38)	< 0.0001

### Co-IRs expressions of T cell activation and exhaustion between SLE and healthy controls

The functionality of T cells is strictly controlled by an abundance of immune modulating signals from immunological inhibition and activation surface molecules, that include HLA-DR, CD38, inducible costimulatory molecule (ICOS), TIGIT, PD-1 and T cell immunoglobulin, and mucin domain-containing protein 3 (TIM3). In addition to upregulating PD-1, exhausted T cells lose the capacity to differentiate into memory cells, as indicated by the expression of the interleukin-7 receptor (IL-7R; CD127). On CD4 + and CD8 + T cells, we evaluate the frequency and intensity of T cell activation (CD38 and HLA-DR) and several Co-IRs, including TIGIT, PD-1, CD127, CD160, signaling lymphocytic activation molecule family member 4 (SLAMF4; CD244; 2B4) and TIM3 expression. Table 3 showed that the percentages of CD279 (8.027 ± 0.7983%, (*N* = 91) vs. 3.93 ± 0.7035%, (*N* = 27), *p* = 0.008) and TIGIT (25.67 ± 1.119%, (*N* = 90) vs. 20.13 ± 0.8377% (*N* = 26), *p* = 0.0109) expressions on CD4^+^ T cells were expanded in SLE patient. In contrast, CD127 expression on CD4 + T cells was reduced in SLE patients compared to HC (33.85 ± 2.343%, (*N* = 90) vs. 44.59 ± 5.149% (*N* = 27), *p* = 0.0382; MFI: 394.1 ± 6.585 vs. 421.3 ± 12.36, *p* = 0.0439). Also, TIM3 (12.56 ± 0.9711%, (*N* = 91) vs. 9.826 ± 1.19% (*N* = 27), *p* = 0.1534; MFI: 444.3 ± 7.502 vs. 384.9 ± 12.23, *p* = 0.0002), TIGIT (35.06 ± 1.65%, (*N* = 90) vs. 25.81 ± 1.774% (*N* = 27), *p* = 0.0042) and HLA-DR (46.74 ± 2.319% (*N* = 51) vs. 18.6 ± 1.909% (*N* = 17), *p* < 0.0001; MFI: 740.2 ± 31.93 vs. 547.2 ± 24.81, *p* = 0.0013) expression on CD8^+^ T cells were higher in patients with SLE. In contrast, CD127 expression on CD8 + T cells was reduced in SLE patients compared to HC (22.46 ± 1.752%, (*N* = 90) vs. 40.86 ± 4.19% (*N* = 27), *p* < 0.0001; MFI: 360.8 ± 7.882 vs. 421 ± 14.87, *p* = 0.0003). CD160 expression on CD3 + T cells was higher in SLE patients compared to HC (16.92 ± 1.199, (*N* = 91) vs. 10.93 ± 1.137, (*N* = 27) *p* = 0.0101). However, CD160 MFI expression on CD8 + T cells was lower in SLE patients compared to HC (MFI: 717.8 ± 14.43, (*N* = 86) vs. 791.4 ± 21.65, (*N* = 26) *p* = 0.0121). As shown in Table 4, CD8 + CD279 + TIM3 + (0.5922 ± 0.09517, (*N* = 90) vs. 0.08077 ± 0.01666, (*N* = 26), *p* = 0.0048) and CD8 + HLA-DR + CD38 + (13.75 ± 1.162 (*N* = 87), vs. 4.519 ± 0.8416 (*N* = 27), *p* < 0.0001) T cells were higher in patients with SLE.

**Table 3:** CD4 and CD8 T cell expression of cell single activation and Co-IRs in SLE and normal

Markers	Mean ± SD (percentage)		*P* Value
	SLE	Normal	
CD4 + CD279 +	8.027 ± 0.7983 (91)	3.93 ± 0.7035 (27)	0.008
CD4 + TIM3 +	4.869 ± 0.5852 (91)	4.015 ± 0.9389 (26)	0.4801
CD4 + CTLA4 +	0.2584 ± 0.06174 (89)	0.08889 ± 0.0209 (27)	0.1363
CD4 + LAG3 +	1.013 ± 0.1755 (91)	0.84 ± 0.1783 (25)	0.6201
CD4 + CD127 +	33.85 ± 2.343 (90)	44.59 ± 5.149 (27)	0.0382
CD4 + TIGIT +	25.67 ± 1.119 (90)	20.13 ± 0.8377 (26)	0.0109
CD8 + CD279 +	7.718 ± 0.8665 (91)	4.896 ± 0.9672 (27)	0.0955
CD8 + TIM3 +	12.56 ± 0.9711 (91)	9.826 ± 1.19 (27)	0.1534
CD8 + CTLA4 +	0.2225 ± 0.03828 (89)	0.163 ± 0.03626 (27)	0.4132
CD8 + LAG3 +	0.5511 ± 0.1479 (90)	0.1963 ± 0.05324 (27)	0.1954
CD8 + CD127 +	22.46 ± 1.752 (90)	40.86 ± 4.19 (27)	< 0.0001
CD8 + TIGIT +	35.06 ± 1.65 (90)	25.81 ± 1.774 (27)	0.0042
CD8 + CD160 +	33.69 ± 1.574 (89)	29.04 ± 1.988 (27)	0.1322
CD8 + CD244 +	1.634 ± 0.358 (88)	1.726 ± 0.4429 (27)	0.8947
CD8 + HLA-DR +	46.74 ± 2.319 (51)	18.6 ± 1.909 (17)	< 0.0001
CD8 + CD38 +	25.92 ± 1.962 (51)	22.97 ± 2.123 (17)	0.4186
CD3 + CD160 +	16.92 ± 1.199 (91)	10.93 ± 1.137 (27)	0.0101
CD3 + CD244 +	5.79 ± 1.11 (91)	7.477 ± 2.673 (26)	0.5041
CD3 + CD279 +	6.824 ± 0.6774 (91)	3.978 ± 0.7626 (27)	0.0323
CD3 + TIGIT +	32.06 ± 1.643 (91)	28 ± 2.888 (27)	0.235
CD3 + NKG2C +	8.953 ± 1.342 (91)	9 ± 2.623 (26)	0.9869

**Table 4:** CD4 and CD8 T cell expression of cell double activation and Co-IRs in SLE and normal

Markers	Mean ± SD (percentage)		*P* Value
	SLE	Normal	
CD4 + CD279 + TIM3 +	0.6607 ± 0.09546 (89)	0.3667 ± 0.0859 (27)	0.1052
CD4 + CTLA4 + LAG3 +	0.1733 ± 0.04774 (90)	0.07037 ± 0.02123 (27)	0.2454
CD4 + CD127 + TIGIT +	4.133 ± 0.3952 (90)	5.041 ± 0.6587 (27)	0.2632
CD8 + CD279 + TIM3 +	0.5922 ± 0.09517 (90)	0.08077 ± 0.01666 (26)	0.0048
CD8 + CTLA4 + LAG3 +	0.01429 ± 0.004835 (91)	0.007407 ± 0.005136 (27)	0.4621
CD8 + CD127 + TIGIT +	2.266 ± 0.2667 (90)	2.426 ± 0.4004 (27)	0.7647
CD8 + CD160 + CD244 +	0.5455 ± 0.1769 (88)	0.8556 ± 0.3153 (27)	0.3961
CD8 + HLA-DR + CD127 +	1.923 ± 0.3112 (53)	1.716 ± 0.3932 (19)	0.7185
CD8 + HLA-DR + CD38 +	13.75 ± 1.162 (87)	4.519 ± 0.8416 (27)	< 0.0001
CD3 + CD160 + CD244 +	0.4652 ± 0.06847 (89)	0.2423 ± 0.06255 (26)	0.0927
CD3 + CD279 + TIGIT +	4.089 ± 0.4359 (91)	2.804 ± 0.5352 (27)	0.1346

### Co-IRs expression on T cell correlated to SLE clinical disease parameters

**Fig. 4:**
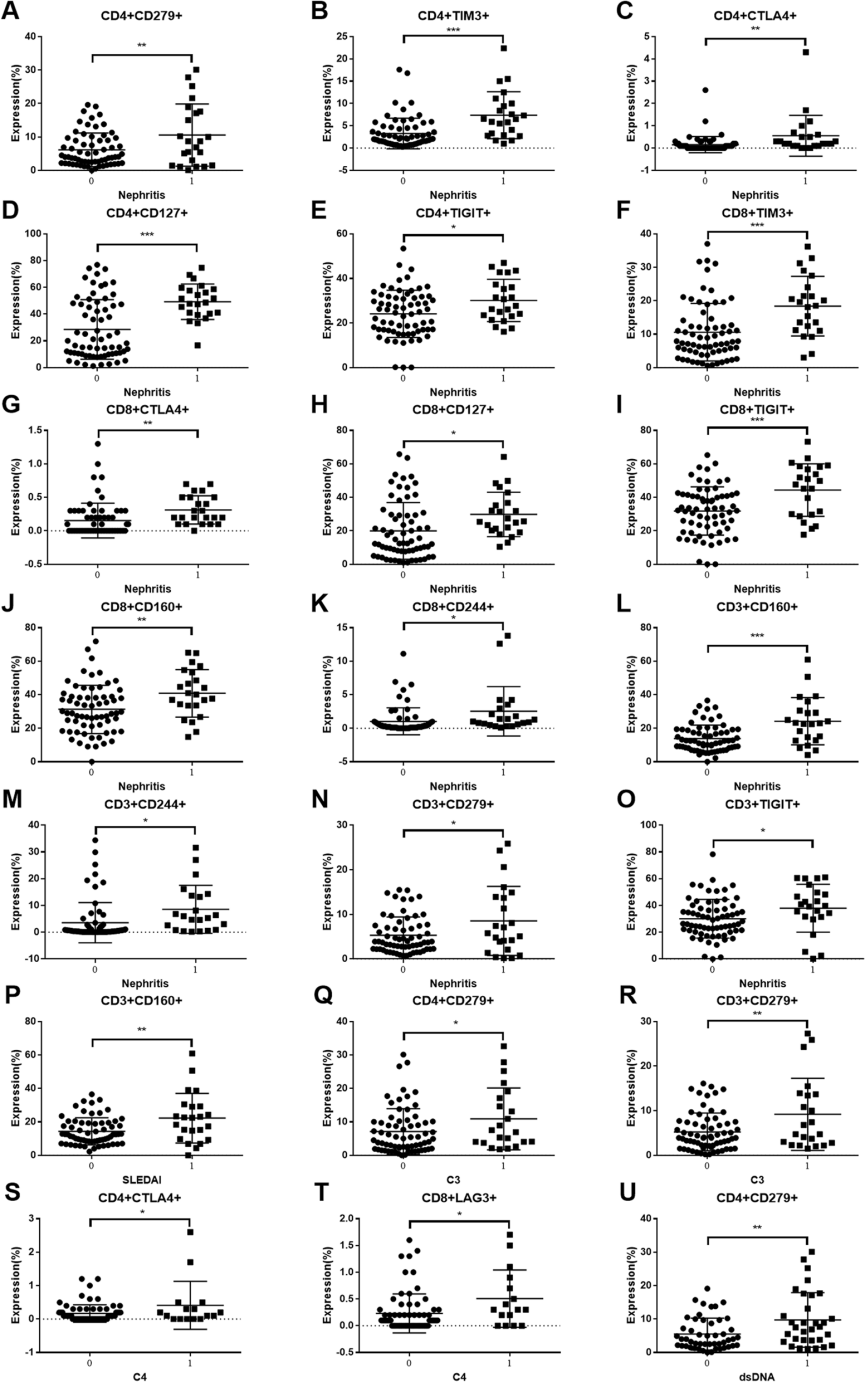
The performance of Co-IRs expression on T cell correlation with a series of clinical manifestations, disease activity and laboratory features including presence of nephritis (proteinuria < 0.5gm vs. > 0.5gm), decreased complement component 3 (C3) and/or complement component 4 (C4), disease activity (SLEDAI > 6) and ds-DNA antibody production. The *p* values presentation as above Fig. 3

We next determined several Co-IRs and activation markers including TIGIT, PD1, TIM3, CD160, HLA-DR, CD38 and CD127 expression on CD3^+^/CD4^+^/CD8^+^ T cells and analyzed the performance of their correlation with a series of clinical manifestations, disease activity and laboratory features including presence of nephritis (proteinuria < 0.5gm vs. > 0.5gm), decreased complement component 3 (C3) and/or complement component 4 (C4), disease activity (SLEDAI > 6) and ds-DNA antibody production. Figure 4A to E showed SLE patients with nephritis exhibited higher frequencies of CD4^+^ T cells expressing PD1, CTLA4, TIM3, CD127 and TIGIT [CD4 + CD279 + (6.162 ± 0.6218%, (*N* = 65) vs. 10.57 ± 1.938%, (*N* = 23), *p* = 0.0056); CD4 + CTLA4 + (0.1545 ± 0.04463%, (*N* = 66) vs. 0.5565 ± 0.1915%, (*N* = 23), *p* = 0.0038); CD4 + TIM3 + (3.279 ± 0.4234%, (*N* = 66) vs. 7.378 ± 1.095%, (*N* = 23), *p* < 0.0001); CD4 + CD127 + (28.55 ± 2.72%, (*N* = 67) vs. 49.3 ± 2.767%, (*N* = 23), *p* < 0.0001); CD4 + TIGIT + (24.13 ± 1.297%, (*N* = 67) vs. 30.14 ± 1.978%, (*N* = 23), *p* = 0.0184)]. CD8^+^ T cells (Fig. 4F to K) express higher TIM3, CTLA4, CD127, TIGIT, CD160 and CD244 [CD8 + TIM3 + (10.59 ± 1.041%, (*N* = 68) vs. 18.38 ± 1.858%, (*N* = 23), *p* = 0.0003); CD8 + CTLA4 + (0.1538 ± 0.0323%, (*N* = 65) vs. 0.313 ± 0.04415%, (*N* = 23), *p* = 0.0099); CD8 + CD127 + (19.94 ± 2.074%, (*N* = 67) vs. 29.82 ± 2.773%, (*N* = 23), *p* = 0.013); CD8 + TIGIT + (31.86 ± 1.76%, (*N* = 67) vs. 44.38 ± 3.271%, (*N* = 23), *p* = 0.0007); CD8 + CD160 + (31.2 ± 1.77%, (*N* = 66) vs. 40.82 ± 2.944%, (*N* = 23), *p* = 0.0068); CD8 + CD244 + (1.025 ± 0.2473%, (*N* = 65) vs. 2.523 ± 0.7825%, (*N* = 22), *p* = 0.0178)]. SLE patients with high disease activity (SLEDAI < 6 vs. SLEDAI ≥ 6) have expanded cell numbers of CD3 + CD160 + (14.32 ± 1.012%, (*N* = 64) vs 22.22 ± 3.09%, (*N* = 23), *p* = 0.0022). SLE patients with C3 depression have significant higher expression cells number (%) of CD4 + CD279 + (7.109 ± 0.8234%, (*N* = 69) vs 10.91 ± 1.973%, (*N* = 22), *p* = 0.0408) and CD3 + CD279 + (5.245 ± 0.5207%, (*N* = 66) vs 9.191 ± 1.728%, (*N* = 22), *p* = 0.0041). C4 depression showed lower expression of CD4 + CTLA4 + % (0.1681 ± 0.03076, (*N* = 72) vs. 0.4125 ± 0.1793, (*N* = 16), *p* = 0.023 and CD8 + LAG3 + (0.23 ± 0.04366, (*N* = 70) vs. 0.5063 ± 0.134, (*N* = 16), *p* = 0.0149). SLE patients with positive dsDNA demonstrated higher expression cells number (%) of CD4 + CD279 + (5.516 ± 0.6812%, (*N* = 49) vs 9.756 ± 1.44%, (*N* = 32), *p* = 0.0041). The detail of Co-IRs and activation markers correlation with a series of clinical manifestations, disease activity and laboratory features were listed in Supplemental Tables 2 , 3, 4, 5 and 6.

**Fig. 5:**
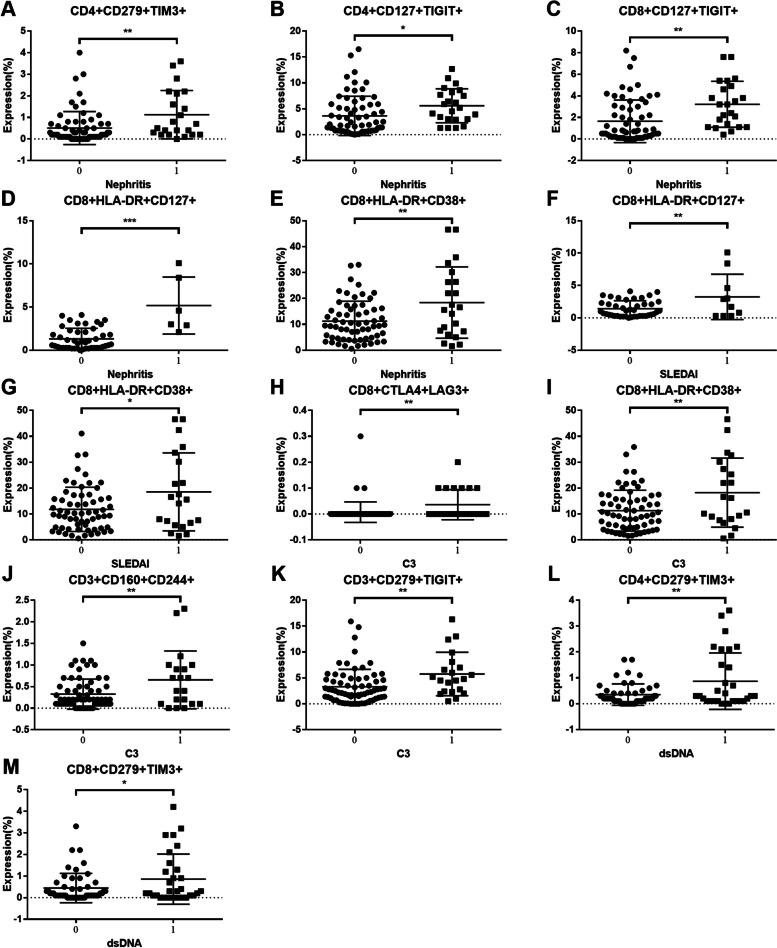
The performance of two Co-IRs expression on T cell correlation with a series of clinical manifestations, disease activity and laboratory features. The *p* values presentation as above Fig. 3

Regarding two Co-IRs analysis, Fig. 5A to E demonstrated nephritis patients exhibited a statistically significant increase in the cell numbers of CD4 + CD279 + TIM3 + (0.5075 ± 0.09351%, (*N* = 67) vs. 1.127 ± 0.2388%, (*N* = 22), *p* = 0.0045); CD4 + CD127 + TIGIT + (3.634 ± 0.4631%, (*N* = 67) vs. 5.587 ± 0.6844%, (*N* = 23), *p* = 0.0303); CD8 + HLA-DR + CD127 + (1.322 ± 0.1797%, (*N* = 46) vs. 5.183 ± 1.346%, (*N* = 6), *p* < 0.0001); CD8 + HLA-DR + CD38 + (11.23 ± 0.9701%, (*N* = 63) vs. 18.41 ± 2.934%, (*N* = 22), *p* = 0.0033). SLE patients with high disease activity (Fig. 5F and G) showed expanded CD8 + HLA-DR + CD38 + (11.81 ± 1.076%, (*N* = 63) vs. 18.55 ± 3.281%, (*N* = 21), *p* = 0.0127; CD8 + HLA-DR + CD127 + 1.417 ± 0.1921%, (*N* = 42) vs. 3.24 ± 1.105%, (*N* = 10), *p* = 0.0076). C3 depression (Fig. 5H to K) had low CD8 + HLA-DR + CD38 + (11.32 ± 0.9845%, (*N* = 64) vs. 18.26 ± 2.912%, (*N* = 21), *p* = 0.0047); CD8 + CTLA4 + LAG3 + (0.007246 ± 0.004762%, (*N* = 69) vs. 0.03636 ± 0.01239%, (*N* = 22), *p* = 0.0092); CD3 + CD160 + CD244 + (0.3254 ± 0.04261%, (*N* = 67) vs. 0.655 ± 0.1496%, (*N* = 20), *p* = 0.0043) and CD3 + CD279 + TIGIT + (3.312 ± 0.4021 (69) vs. 5.757 ± 0.9143 (21), *p* = 0.007). SLE patients with positive dsDNA (Fig. 5L and M) showed higher expression of CD4 + CD279 + TIM3 + (0.3531 ± 0.059%, (*N* = 49) vs. 0.87 ± 0.199%, (*N* = 30), *p* = 0.0037).

**Fig. 6:**
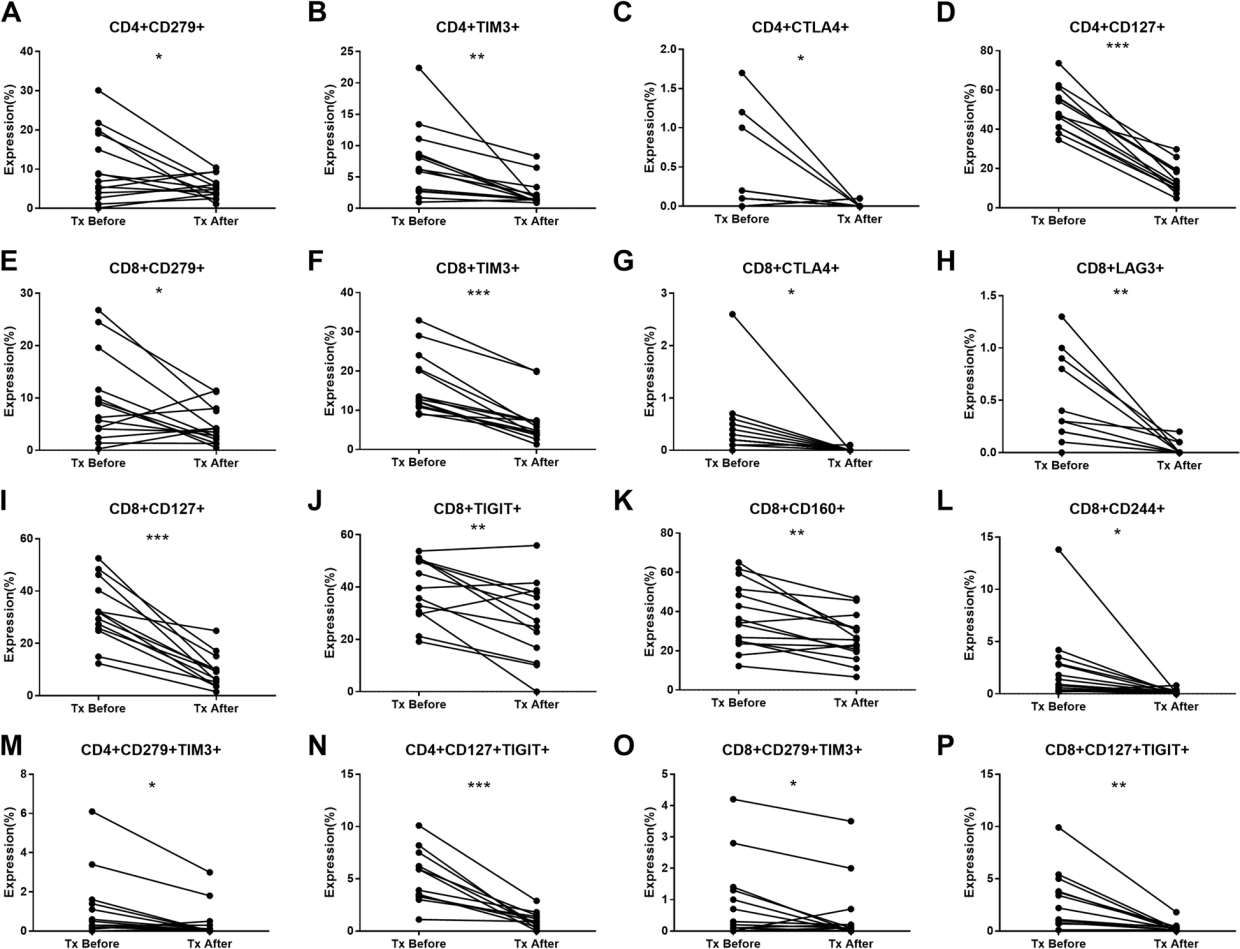
The performance of Co-IRs expression on T cell changes after treatment. The *p* values presentation as above Fig. 3

Subsequently, we compared the levels of activated (CD38, HLA-DR), functioning (CD127), and exhaustion (PD-1, CTLA4, TIGIT, Tim-3, CD160, and CD244) markers on T cells prior to and following seven to twenty-three moths immunotherapy. Notably, higher Co-IRs expression levels of most SLE patients decreased after treatment (Fig. 6), indicating that Co-IRs monitor are useful tools in determining the treatment response, including CD4 + CD279 + (*p* = 0.0405, *N* = 14), CD4 + TIM3 + (*p* = 0.0043, *N* = 14), CD4 + CTLA4 + (*p* = 0.0494, *N* = 13), CD4 + CD127 + (*p* < 0.0001, *N* = 13), CD8 + CD279 + (*p* = 0.0317, *N* = 14), CD8 + TIM3 + (*p* < 0.0001, *N* = 14), CD8 + CTLA4 + (*p* = 0.0371, *N* = 13), CD8 + LAG3 + (*p* = 0.0061, *N* = 12), CD8 + CD127 + (*p* < 0.0001, *N* = 13), CD8 + TIGIT + (*p* = 0.0034, *N* = 13), CD8 + CD160 + (*p* = 0.0024, *N* = 14), CD8 + CD244 + (*p* = 0.0305, *N* = 14), CD4 + CD279 + TIM3 + (*p* = 0.0159, *N* = 13), CD4 + CD127 + TIGIT + (*p* = 0.0002, *N* = 12), CD8 + CD279 + TIM3 + (*p* = 0.037, *N* = 14), CD8 + CD127 + TIGIT + (*p* = 0.0027, *N* = 12). Our research suggests that abnormal immune activation with Co-IRs expression may contribute to the immune dysregulation observed in SLE disease courses. The detail information of fourteen patients were listed in Supplemental Table 7.

### Soluble PD-1, PDL-2 and TIM3 levels in SLE

**Fig. 7:**
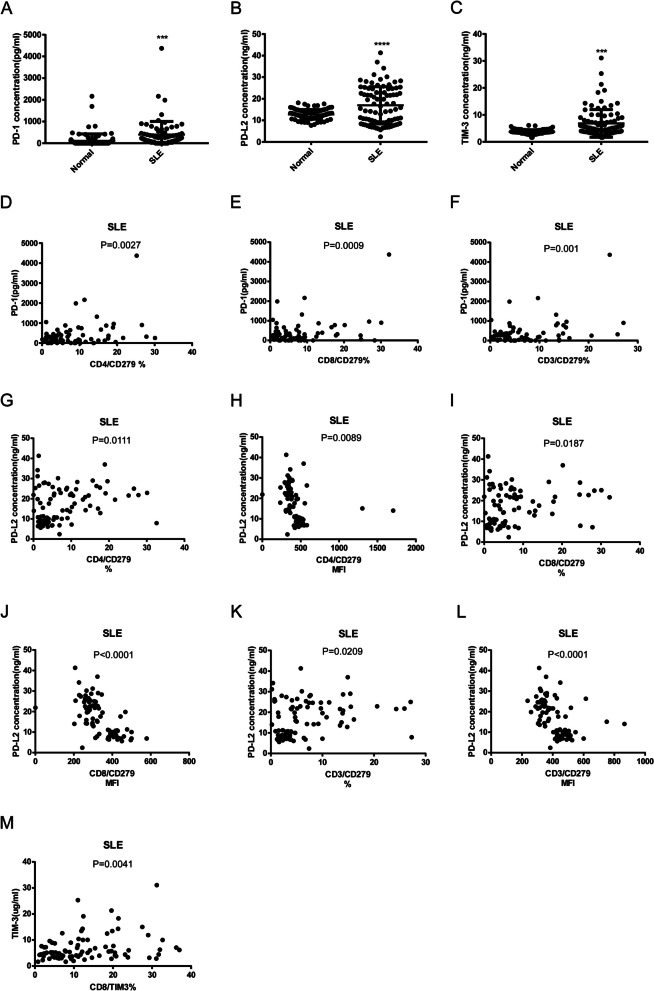
Soluble PD-1, PDL-2 and Tim3 serum levels showed significant elevated in SLE patients and correlation with the performance of Co-IRs expression on T cell. The p values presentation as above Fig. 3

**Fig. 8:**
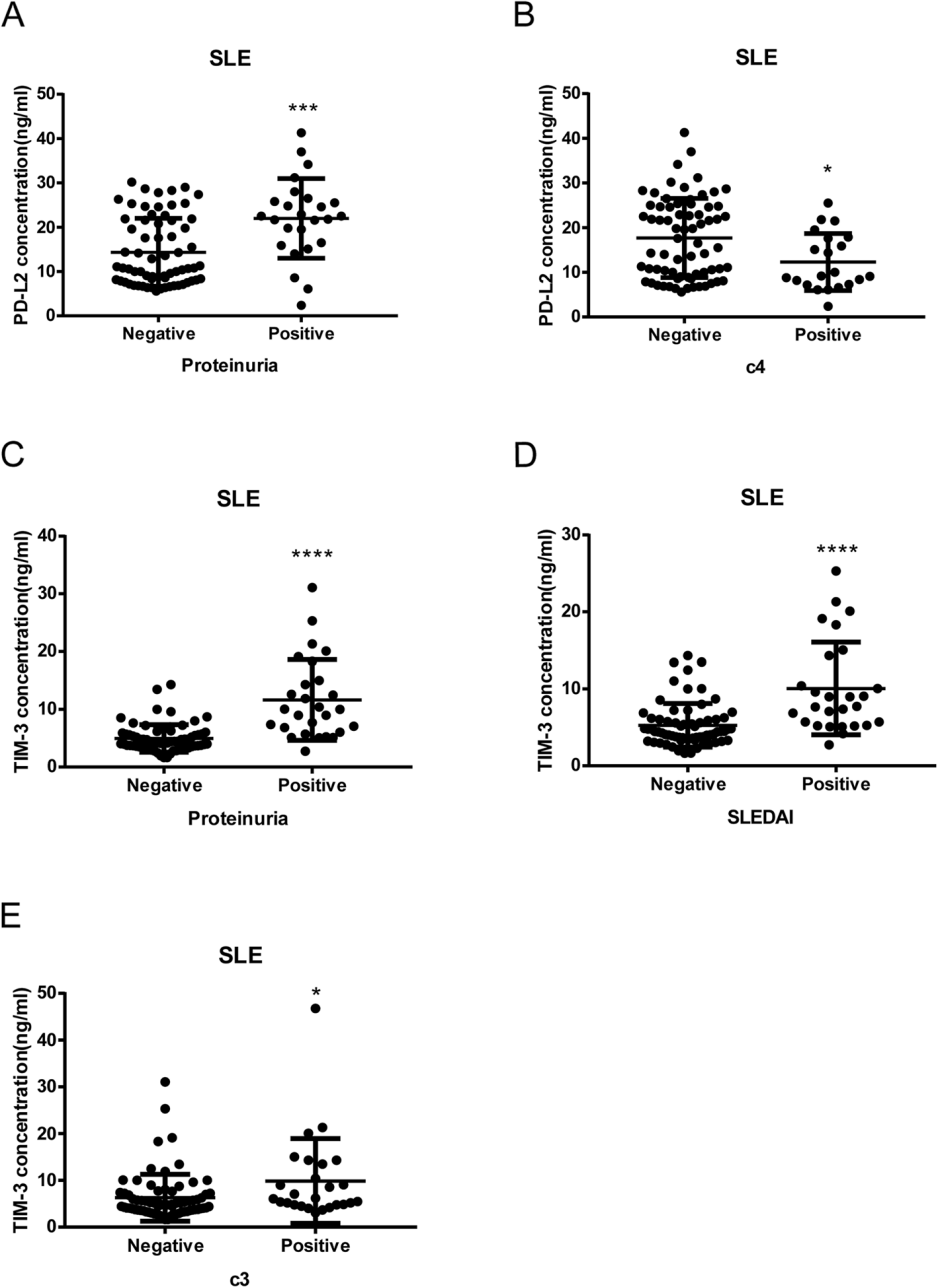
Soluble PD-1, PDL-2 and Tim3 serum levels showed correlation with the presence of nephritis (proteinuria < 0.5gm vs. > 0.5gm), decreased complement component 3 (C3) and/or complement component 4 (C4), and disease activity (SLEDAI > 6). The *p* values presentation as above Fig. 3

PD-L2 is one of the ligands of PD-1 expressed by T cells, and its binding to PD-1 blocks activation signals from the T cell receptor and CD28 in typical T cells. TIM3 modulates Th1 immunity through eliciting apoptosis, prompts the generation of a disintegrin and metalloproteinase, and limits autoimmunity [17]. We then asked whether these soluble mediators can correlate to disease phenotypes. As shown in Fig. 7, SLE patients have significant elevated serum levels of soluble PD-1, PDL-2 and TIM3 than HCs (sPD-1: 396.2 ± 69.71, *N* = 77 vs. 111.1 ± 32.92, *N* = 92 *p* = 0.0001; sPDL2: 16.97 ± 0.9238, *N* = 87 vs 12.78 ± 0.2314, *N* = 92 *p* < 0.0001; sTIM3: 6.822 ± 1.5367, *N* = 91 vs 3.675 ± 0.08441, *N* = 92 *p* < 0.0001). Figure 8 indicated sPD-1 levels were correlated to CD4 + CD279 + % (*p* = 0.0027, *N* = 75), CD8 + CD279 + % (*p* = 0.0009, *N* = 75) and CD3 + CD279 + % (*p* = 0.001, *N* = 75). sPD-L2 levels were correlated to CD4 + CD279 + (% *p* = 0.0111, MFI = 0.0089, *N* = 87), CD4 + CD279 + (% *p* = 0.0187, MFI < 0.0001, *N* = 87) and CD3 + CD279 + (% *p* = 0.0209, MFI =  < 0.0001, *N* = 87). sTIM3 levels were correlated to CD8 + TIM3 + % (*p* = 0.0041, *N* = 90). High sPD-L2 levels correlated with SLE proteinuria (14.37 ± 0.9327, (*N* = 67) vs 21.99 ± 1.797, (*N* = 25), *p* < 0.0001) whereas inverse to C4 depression (17.69 ± 1.055, (*N* = 71) vs 12.31 ± 1.392, (N = 21), *p* = 0.0116). High sTIM3 levels correlated with SLE proteinuria (4.966 ± 0.2901, (*N* = 68) vs 11.63 ± 1.341, (*N* = 27), *p* < 0.0001; high (SLEDAI ≥ 6) disease activity (5.274 ± 0.344, (*N* = 68) vs 10.06 ± 1.157, (*N* = 27), *p* < 0.0001 and C3 depression (6.294 ± 0.587, (*N* = 73) vs 9.872 ± 1.778, (*N* = 26), *p* = 0.0148. They functioned as serologic indicators of disease activity and organ involvement.

### Type I interferon (IFN-β) and JAK inhibitor (JAKi) effects on SLE T cells

**Fig. 9:**
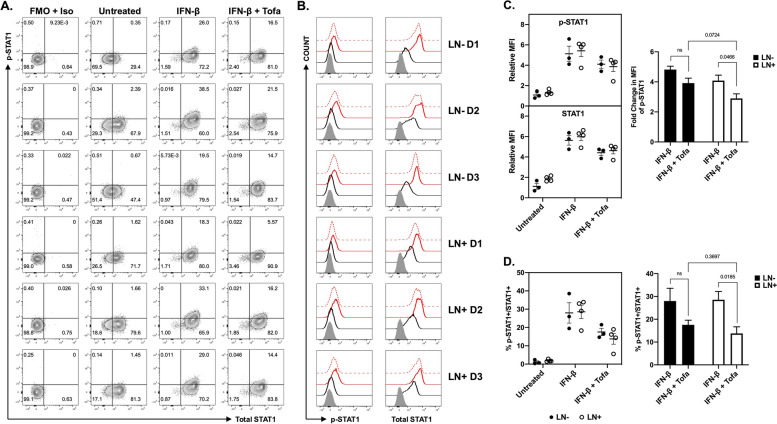
Flow cytometry analysis of phospho and total STAT1 in CD3 + lymphocytes from SLE-LN- and SLE-LN + patients. **A** Prominent proportions of total STAT1 + or p-STAT1 + double-positive lymphocytes were collected from three LN- and three LN + patients were underwent stimulation for 48 h with 200 ng/mL IFN-$\upbeta$ in the presence or absence of 200 nM Tofacitinib. **B** Representative histograms of the levels of p-STAT1 and total STAT1 in LN- and LN + lymphocytes treated, as described in (**A**). The unstimulated cell populations are represented by the solid black lines, whereas the cell populations that were treated with IFN-β and IFN-β + Tofacitinib were denoted by the solid and dashed red lines, respectively. Histograms that were gray-filled depict negative controls (FMO with isotypes). **C** Comparative statistical evaluation of p-STAT1 and total STAT1 relative MFI in B. Bar graphs show the fold change MFI of p-STAT1 in IFN-$\upbeta$-stimulated CD3 + T cells with or without JAKi Tofacitinib between LN- and LN + patients. The results were found to be statistically significant using an unpaired t-test, and the data are presented as mean ± SEM. **D** The percentages of p-STAT1 + /STAT1 + double positive T cells (CD3 +) from LN- and LN + patients with or without IFN-$\upbeta$ stimulation in the presence or absence of Tofacitinib. An unpaired t-test was employed to assess the difference between the two groups, and statistical bar graphs with mean ± SEM were utilized for presenting the quantitative results

The transcriptional regulation of Co-IR expression such as TIGIT, PD-1, TIM-3, LAG-3, and others can be controlled by the Type I IFN-JAK-STAT axis pathway. In an effort to elucidate the Co-IR expression discrepancy found in our study, we applied exogenous IFN-β together with or without the JAK inhibitor Tofacitinib to the PMBC cultures obtained from LN- (*n* = 3) and LN + (*n* = 4) SLE patients. Following stimulation, we employed the FACS analysis to examine the expressions of co-IRs on CD4 + or CD8 + T cell subsets of LN- and LN + patients, including TIGIT, CD38, HLA-DR, CD366, CD279 and CD38 + DR + , as well as the levels of phosphorylated STAT1 (Tyr701) and total STAT1 in CD3 + T cell subsets. As shown in Fig. 9, in response to IFN-β stimulation, both LN- and LN + derived PBMCs exhibited a comparable range of percentages for p-STAT1 + /STAT1 + double positive CD3 + T cells (19.5% to 38.5% in LN- vs 18.3% to 33.1% in LN +). Furthermore, the relative MFI of p-STAT1 (Tyr701) and total STAT1 in these PBMCs increased by four to sixfold in comparison to unstimulated T cells (Fig. 8C). More importantly, we revealed that the CD3 + T cell subsets from LN + patients exhibited a greater degree of responsiveness to Tofacitinib than of those from LN- patients (Fig. 8C and D). With the exception of CD279 on CD4 + T cells, the expression of the majority of co-IRs does not differ significantly in response to IFN- (Supplementary Fig. 4). It is worth noting that LN- patients exhibit reduced levels of CD38 expression, whereas CD38 expression surged following IFN-β induction and declined after JAKi addition. Tofacitinib is more likely to inhibit the expression of CD38 on CD4 + or CD8 + subsets, as well as the expression of CD38 + DR + on CD8 + subsets; therefore, the preponderance of hyperactive p-STAT1 signaling in LN + T lymphocytes might occur in these subpopulations.

## Discussion

The molecular basis of SLE is obscure due to its heterogeneity; however, a combination of genotyping with gene networks, mRNA sequencing, and cellular phenotyping analysis may detect distinct signatures conferring susceptibility to SLE, disease activity, and disease severity [18–20]. Using multidimensional cytometry and transcriptomics to identify SLE-specific phenotypes, this study investigated the unmet need for optimal personalized therapy for SLE. Due to their ability to coordinate and facilitate B cells in promoting autoantibody production, T cells were identified as a key factor in the development of SLE. Several phenotypic and physiological modifications in T cell populations that increase the probability of lupus-related inflammation are being identified [21]. SLE patients with ESRD exhibited a novel transcriptional and phenotypic profile with minimal expression of cytotoxic granules, CD38, and HLA-DR on CD4 + and CD8 + T cells. Long-term, continuous antigenic priming generated specific subsets of exhausted CD4 + and CD8 + T cells that promote functional T cell silencing of lupus nephritis. The flaws of T cells cytotoxicity in SLE patients explain not only the origin of autoimmunity due to the inability to eradicate autoreactive B cells but also the markedly reduced antiviral responses [22] that contribute to uncontrolled Epstein-Barr virus infection [23–25] and EBV reactivation in SLE disease activity [26].

Several identified genes are indispensable for cytotoxicity, signaling, and inflammatory factor production. NKG7 is necessary for cytotoxic degranulation in their mobilization and transport of perforin and GZMB cytotoxicity granules comprising vesicles, which entails the transfer of CD107a to the cell surface and the eradication of targeted cells [27]. FCRL6 possesses inhibitory properties with cytosolic cysteine-rich motif and engages SHP-2 through phosphorylation of ITIM. It interacts extracellularly with MHCII/HLA-DR and is uniquely expressed on cytotoxic T and NK cells [28–30]. In a cross-ancestry meta-analysis, pleckstrin homology domain containing family F member 2 gene (PLEKHF2) loci was identified as a functional locus related to IFN-α production in dendritic cells and NK cells in patients with SLE [31]. ITGAM modulates the immune functions of CD8 T cells and macrophages [32].

CD8 T cells promote lupus disease activity through generating IFN-γ and directly inducing tissue damage. CD8 T cells produce an excess of perforin and GZMB, but their responsiveness is reduced. CD8 + cytotoxic T lymphocytes protect against lupus-like disorder by eliminating activated autoreactive B cells via perforin-mediated killing [33]. In diseased kidneys, cytotoxic CD8 + T cells that express high levels of GZMB, perforin, or GZMK have been identified [34]. We observed that CD8^+^ T cells cytotoxic signatures (GZMB, GZMH, granulysin, perforin, IFN-γ, FcγRIII, KIRs and CD94) downregulation may contribute to alternations with poor cytotoxic capacity (Fig. 2), whereas FcγRIII (3A/3B), TBX21, LYN, CCL4L1, and CMKLR1 decrease production in CD8 + T cell, indicating a potentially central role in inactive inflammatory pathways and cell trafficking (Supplemental Figures). SLE with ESRD is associated with a significant loss of functional specific immune responses. Immune senescence markers PD-1 and CD57 did not differ in our two distinct SLE phenotypes, whereas GZMK transcript increased in ESRD indicating potential age-associated GZMK-expressing CD8^+^ T (Taa) cells of exhaustion and tissue homing, which address potential immune system dysfunctions [35]**.** The elevated numbers of CD8 + CD161-GZMK + T cells and CD8 + CD161-GZMK + GZMB + T cells observed in patients with ESRD LN may be major contributing variables. Additional analysis proposed predominant migratory cells to inflammatory tissue are MAIT cells [36] that are CD8 + CD161 + GZMK + , whereas ESRD patients exhibited diminished tissue damage accompanied by the circulation of GZMK + T cells. These novel findings revealed impaired cytotoxicity, dysfunctions of effector T cells and potential immune senescent modulation in end stage LN. The findings of GO and pathway enrichment analyses support the notion that dysfunctions in cytotoxicity, antigen processing and presentation, and chemokine-cytokine pathways are major risk factors. The present study identified the key genes, revealing potential targets for predicting the disease progression and infectious risk of LN. Nonetheless, SLE has a complex blood transcriptome due to heterogeneous cellular origins, and single-cell RNA sequencing (scRNA-seq) that resolves the SLE transcriptional signatures heterogeneity origination could eventually result in precision medicine implementations [37].

Higher Co-IRs expressions on T cells might be related to T cell exhaustion, in which dysfunctional effector cytotoxicity reactions are gradually extinguished to prevent noteworthy collateral tissue damage as a fundamental concept of T cell dysfunction that evolved to limit immunopathology. CD8 + T cells that are exhausted are unable to eliminating their intended targets. Global exhaustion signatures in CD8 + T cells have been coupled with a long-term disease silence and beneficial responses to therapy in lupus patients [38]. In exhausted T cells, the genes 4–1BB (CD137), CTLA4, PD-1, Leukocyte immunoglobulin-like receptor subunit B member 4 (LILRB4), and KLG1 are upregulated [39]. Variable PD-1 and ICOS co-expression T cells are elevated, whereas PD1 + ICOS + Tem cells correlate with SLE disease progression and are encompassed by exhausted T cells, which correspond to a lupus-silent course [40]. In lupus-affected mice, kidney-infiltrating T cells become activated effector cells that cause injury to tissues and, eventually, failure of the organs. Tissue parenchyma is able to suppress T-cell responses and limit self-damage [41]. Our research identified high expressions of PD1, CTLA4, TIM3, and TIGIT on T cells were correlated with disease activity, nephritis, and treatment response downregulation in SLE. T cell-depleted Co-IRs execute a dynamic role in effector T cells differentiation throughout the heterogeneous course of SLE.

We observed an increase in CD3 + CD160 + CD244 + T cells in SLE patients with persistent proteinuria, high disease activity, and C3/C4 depression. SLE patients had decreased levels of SLAMF7 and SLAMF4 (CD244) on memory CD8 + T cells, suggesting deficient antiviral effector function with inadequate effector CD8 + T cell degranulation capacity and a proportion of IFN-producing cells in response to antigen stimulation [42, 43]. The expression of SLAMF4 on monocytes was diminished in patients with SLE, which inversely associated with serum autoantibody quantities [44]. Substantial reduced SLAMF4 + CD8 + T cells were detected in SLE patients, resulting in weakened cytotoxic capacity and an impaired ability to combat infection [43, 45]. The inhibition of CD160 to CD160-ligand coupling revived CD8 T-cell proliferation, and the degree of restoration was proportional to the ex vivo CD160 + CD8 T cells, demonstrating that CD160-associated CD8 T-cell dysfunction exists independently of PD-1 expression [46].

CD38, HLA-DR, and CD127 (IL-7R) are distinct markers of chronically activated T-cell phenotypes. T-bet, RUNX3, and EOMES are attenuated in CD8 + CD38 + T cells from SLE patients, resulting in a decrease in CD8 T cell-mediated cytotoxicity and an increase in susceptibility to infection [47]. The CD8 + HLA-DR + CD38 + T cells that have been linked in this study to LN, C3 depression, and SLE disease activity are believed to be the cause of SLE's persistent immune activation. Hyper-activated HLA-DR + CD38 + T cells facilitate viral persistence and loss of immunologic competence in chronic infections. In SLE, CD38 expression in T cells generates Th1 and Th2 inflammatory cytokines, which are correlated with disease activity [48] but may be exhausted for predicting an improved prognosis in lupus [49]. In contrast, transcriptional analysis of blood pathological CD8 + T cells from patients with autoimmune disease indicated a correlation between an activated, non-exhausted CD8 + T cell phenotype and a poor prognosis [49, 50]. We hypothesize that SLE with end-organ injury is associated with a loss of CD38 + HLA-DR + T cells with chronic immune activation. In SLE, type I interferon may increase CD38 levels, whereas JAKi inhibits T cell activation via the CD38 pathway, according to the in vitro study. These results suggest that monitoring T cell exhaustion and cytotoxicity status is beneficial for assessing renal damage and infection risk. Our findings support the need to devise strategies that enhance T cell exhaustion.

SLE patients exhibiting hematologic manifestations and positive anti-dsDNA antibodies showed higher proportions of HLA-DR + T cells and ICOS + T cells [51]. The expression of HLA-DR + T cells had a positive correlation with SLEDAI, and number of TIGIT + T cells was reduced in patients with renal involvement [51]. HLA-DR, costimulatory molecules on activation-related circulating T cell subsets, and relevant chemokines and cytokines all contribute to the onset of SLE. In SLE patients, we observed a decrease in CD127, whereas CD127 + TIGIT + T cells have a strong correlation with therapeutic responses. CD127 + memory T cells suppress CD244 and cytotoxic granules expression [52]. CD127 restitution and TIGIT co-expression on nascent CD4 + and CD8 + memory T cells [49] correlate with the reduction of pathogenic T cell subsets and are valuable for assessing and predicting lupus treatment efficacy.

Following activation, diverse costimulatory and co-inhibitory molecules are dynamically expressed on the surface of T cells. ICOS is a costimulatory receptor, whereas PD-1, TIGIT, and TIM3 are Co-IRs that inhibit CD4 + and CD8 + T cell responses. PD-1 is extensively expressed on a wide variety of T cell subtypes. Circulating PD-1 + ICOS + Tfh, PD-1 + ICOS + Tcm, and PD-1 + ICOS + Tem were all significantly increased in patients with SLE [40, 53]. Additionally, CXCR5-CXCR3 + PD-1^hi^ CD4 + helper T cells were observed [54], as were CXCR5^hi^ ICOS^hi^ PD-1^hi^ Tfh-like T cells [55]. PD-1 + CXCR5-CD4 + T peripheral helper (Tph) cells correlated with SLE disease parameters and progress of the disease [56]. The investigation into the potential efficacy of the PD-1-PD-L1 pathway as an inhibitory mechanism in the progression and development of LN in mice, and the search for a potential efficient treatment approach for SLE [57]. Significant to the pathogenesis of SLE, two critical signaling pathways, type I interferon and toll-like receptor, influence the expression of PD-1 and its ligands (PD-L1, PD-L2) via activation of NF-κB and/or STAT1 [58]. However, the inability of inhibitory receptors to inhibit the excessive activation of T cells might have been due to the upregulation of alternative activation pathways.

Patients with active SLE who screened confirmed for anti-dsDNA autoantibodies had elevated serum levels of sPD-1 and sPD-L2 [59]. TIM3 have been co-expressed and co-regulated on dysfunctional or 'exhausted' T cells during protracted viral infections and cancers [60]. Using the SLEDAI-2 K score, sTIM3 is associated with disease activity, organ damage, and active renal disease [61]. Expression of TIM3 and co-expression of TIM3 and Fas on particular peripheral T populations has been linked to disease activity in patients with SLE [62]. The bioactivity of sTIM3 and sPD-1 ensures that they maintain the capacity to bind to the respective receptors or ligands. In competition with the ligand, these proteins impede the inhibitory effects of PD-1 and TIM3 signals that are bound to membranes, thereby facilitating T-cell activation [63]. It can be difficult to predict episodes and remission of cyclical diseases like SLE and to develop an accurate biomarker for disease assessment. According to our findings, CD4 + T cells and CD8 + T cells that co-express PD-1 and TIM3 were substantially more prevalent in SLE patients with LN, elevated disease activity, C3 depression, and anti-dsDNA antibodies nevertheless diminished following immunotherapy. Age-associated CD8 + TIM3 + PD-1 + T cells evidenced more prominent signs of exhaustion, proliferation defects in response to either homeostatic or TCR stimulation, and altered cytokine secretion, while producing the immunosuppressive cytokine IL-10 [64]. Throughout chronic infection, virus-specific CD8 T cells preserved strong TIM3 expression, expressed together PD-1, and displayed diminished levels of the effector cytokines IFN-γ, TNF and IL-2 [65]. This population represents the most dysfunctional exhausted cells. These findings suggested that the expression of co-inhibitory receptors is a crucial determinant of autoimmunity. Our research provides vital insights into T cell exhaustion for the creation of a more accurate disease severity prediction profile. These immune cell surface markers could serve as diagnostic biomarkers for SLE, and this specific pathway could be a therapeutic target for SLE. Moreover, our results suggest that the elevated sPD1/PD-L2/Tim3 levels associated with SLE disease activity might be utilized as a therapeutic biomarker for response evaluation.

The levels of TIGIT expression on CD4 + T cells increased substantially in SLE patients and had a strong correlation with disease activity [66, 67]; however, activation, proliferation, and production of cytokines were decreased [67]. It was recently reported that type 1 interferon (IFN-I) stimulates LAG-3 expression while inhibiting TIGIT expression on human naive CD4 + and CD8 + T cells [66]. However, no substantial alterations in TIGIT expression were observed upon induction of IFN-β. Furthermore, JAKi provided evidence that the regulation of CO-IRs is carried out by specific transcription networks that are linked to Type I interferon. However, it is conceivable that the lymphocytes of LN + patients employed a unique stimulus to regulate the JAK-STAT pathway, as evidenced by their greater reactivity to Tofacitinib. Treg cells are an additional designation given to CD4 + TIGIT + cells that are co-expressed with Foxp3 and Helio as a result of the effector function of these cells [68, 69]. T cells that are CD8 + TIGIT + which are suggestive of hyperactivated or exhausted T cells, merit additional investigation.

## Conclusion

SLE is a relapsing, refractory disease with limitations due to clinical heterogeneity resulting from cellular, serologic, and other abnormalities. We identify distinct subject subgroups and predict long-term prognosis by establishing a stratification of lupus patients based on a specific transcriptome signature and pathologically altered T cells that lead to active and progressive disease. This research is particularly beneficial in the clinical setting for identifying potentially blood-based markers essential for the initiation of SLE, classifying the extent of disease or predicting disease outcome, and minimizing treatment-related complications.

### Supplementary Information

**Additional file 1: Supplementary Figures 1 to 3. ** The gene list best defining the pathways influence the pathogenesis of LN in long term ESRD. **Supplementary Figure 4.** Co-IRs expressions on CD4+ or CD8+ subsets subsequent to stimulation with IFN-β and JAKi. **Supplementary Table 1. **The clinical characteristics of SLE patients and normal controls. **Supplementary Table 2.**  The comparison one Co-IRs expression on T cells between SLE nephritis negative and positive. **Supplementary Table 3.**  The comparison one Co-IRs expression on T cells between SLE SLEDAI <6 and ≧6. **Supplementary Table 4.** The comparison one Co-IRs expression on T cells between SLE C3 ≧70 and <70. **Supplementary Table 5.** The comparison one Co-IRs expression on T cells between SLE C4 ≧10 and <10. **Supplementary Table 6.** The comparison one Co-IRs expression on T cells between SLE dsDNA <130 and ≧130. **Supplementary Table 7.** The comparison one Co-IRs expression on T cells between SLE before and following therapy.

## Data Availability

The data presented in this study are available on request from the corresponding author.

## Abbreviations

SLE: Systemic lupus erythematosus
LN: Lupus nephritis
ESRD: End renal stage disease
DEG: Differential Expression Gene
PCA: Principal component analysis
PLEK: Platelet and leukocyte C kinase substrate and the KSTR sequence of amino acids
FGFBP2: Fibroblast growth factor binding protein type 2
FGF: Fibroblast growth factor
SLCO4C1: Solute carrier organic anion transporter family member 4C1
FCRL6: Fc receptor-like 6
ITGAM: Integrin subunit alpha M
TBX21: T-box transcription factors
CCL4L1: C–C motif chemokine ligand 4 like 1
CMKLR1: Chemerin chemokine-like receptor 1
CD94: Killer cell lectin like receptor D1
NRCAM: Neuronal cell adhesion molecule
DSEL: Dermatan sulfate epimerase like
TIGIT: T cell immune-receptor with Ig and immune-receptor tyrosine-based inhibitory domains
PD-1: Programmed cell death 1
PD-L2: Programmed death-ligand 2
TIM3: T cell immunoglobulin, and mucin domain-containing protein 3
SLAM4: Signaling lymphocytic activation molecule family member 4
IL7R: Interleukin-7 receptor (CD127)
CXCR3: C-X-C chemokine receptor type 3
ICOS: Inducible T-cell costimulatory
CXCR5: C-X-C chemokine receptor type 5
