# Supplementary material for: T cell expressions of aberrant gene signatures and Co-inhibitory receptors (Co-IRs) as predictors of renal damage and lupus disease activity
Source: J Biomed Sci. 2024 Apr 22;31:41. doi: 10.1186/s12929-024-01024-7 (PMC11034032; doi:10.1186/s12929-024-01024-7)
Supplement: Supplementary file 1 — Additional file 1: Supplementary Figures 1 to 3. The gene list best defining the pathways influence the pathogenesis of LN in long term ESRD. Supplementary Figure 4. Co-IRs expressions on CD4+ or CD8+ subsets subsequent to stimulation with IFN-β and JAKi. Supplementary Table 1. The clinical characteristics of SLE patients and normal controls. Supplementary Table 2. The comparison one Co-IRs expression on T cells between SLE nephritis negative and positive. Supplementary Table 3. The comparison one Co-IRs expression on T cells between SLE SLEDAI <6 and ≧6. Supplementary Table 4. The comparison one Co-IRs expression on T cells between SLE C3 ≧70 and <70. Supplementary Table 5. The comparison one Co-IRs expression on T cells between SLE C4 ≧10 and <10. Supplementary Table 6. The comparison one Co-IRs expression on T cells between SLE dsDNA <130 and ≧130. Supplementary Table 7. The comparison one Co-IRs expression on T cells between SLE before and following therapy. [file 12929_2024_1024_MOESM1_ESM.docx]

**T cell expressions of aberrant gene signatures and Co-inhibitory receptors (Co-IRs) as predictors of renal damage and lupus disease activity**

**Chin-Man Wang^1^, Yeong-Jian Jan Wu^2^, Jian-Wen Zheng^2^, Li Yu Huang ^2^, Keng Poo Tan^+2^, Ji-Yih Chen^+^*^2^**

**Supplementary Figure 1 to 3: The gene list best defining the pathways influence the pathogenesis of LN in long term ESRD.**

**Supplementary Figure 4: Co-IRs expressions on CD4+ or CD8+ subsets subsequent to stimulation with IFN-β and JAKi.**

**Supplementary Table 1: The clinical characteristics of SLE patients & normal controls**

**Supplementary Table 2: The comparison one Co-IRs expression on T cells between SLE nephritis negative and positive**

**Supplementary Table 3: The comparison one Co-IRs expression on T cells between SLE SLEDAI <6 and ≧6**

**Supplementary Table 4: The comparison one Co-IRs expression on T cells between SLE C3 ≧70 and <70**

**Supplementary Table 5: The comparison one Co-IRs expression on T cells between SLE C4 ≧10 and <10**

**Supplementary Table 6: The comparison one Co-IRs expression on T cells between SLE dsDNA <130 and ≧130**

**Supplementary Table 7: The comparison one Co-IRs expression on T cells between SLE before and following therapy**

**Supplementary Figure 1:**


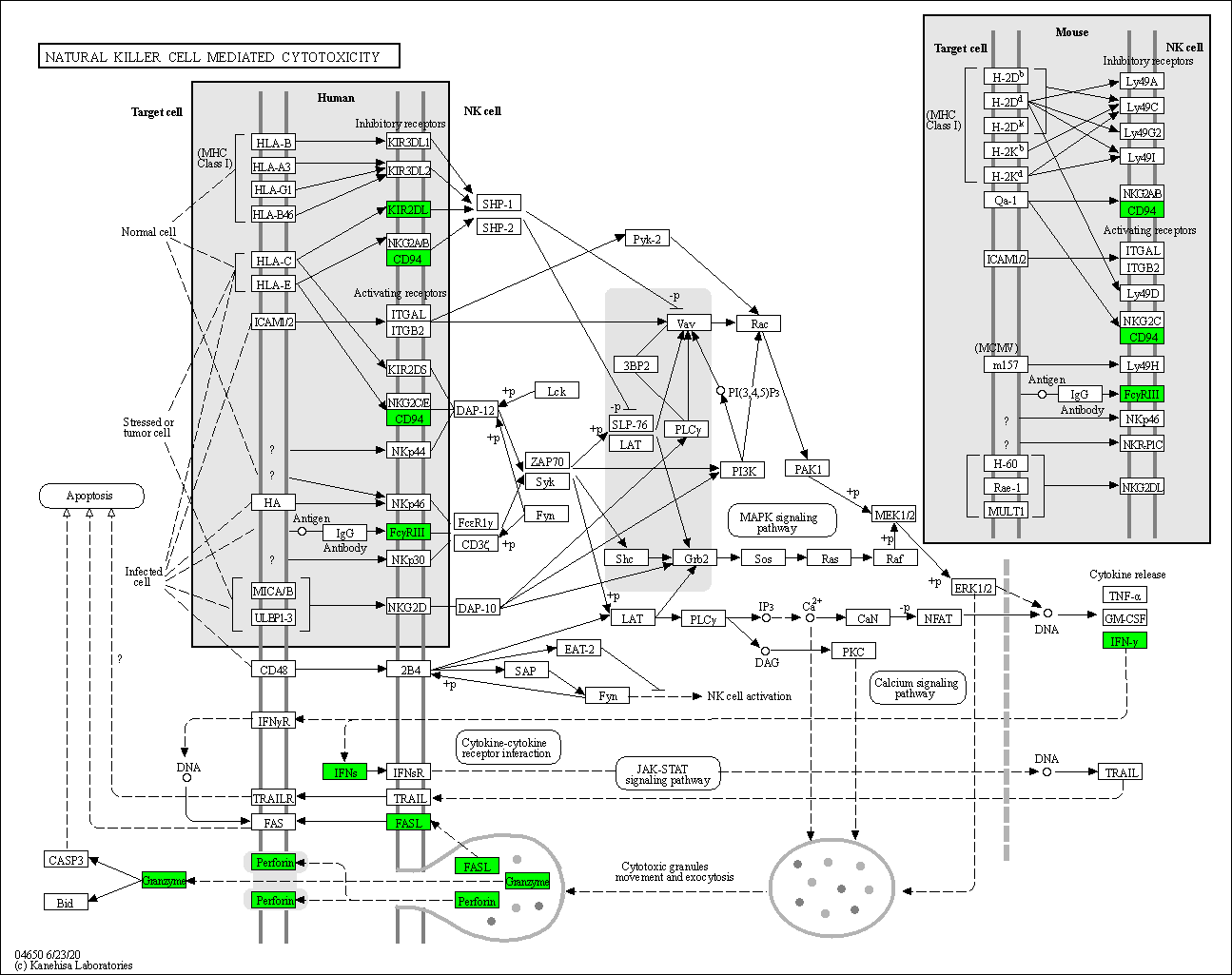


**Supplementary Figure 2:**


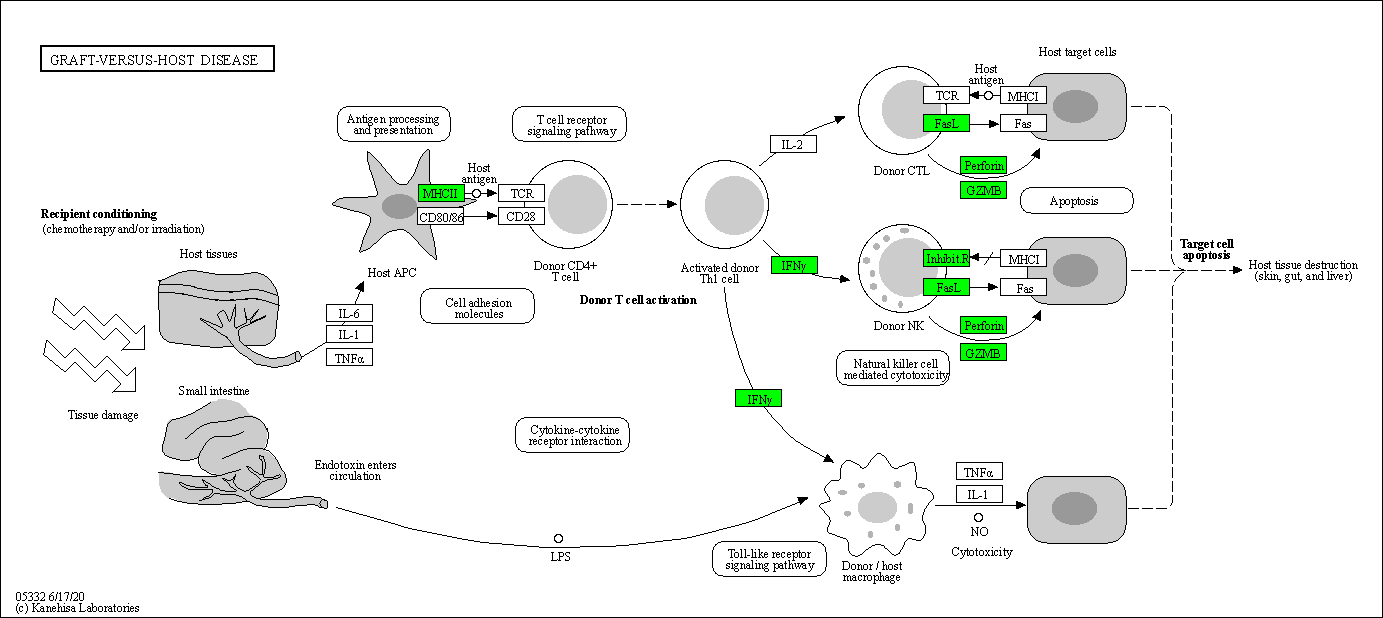


**Supplementary Figure 3:**


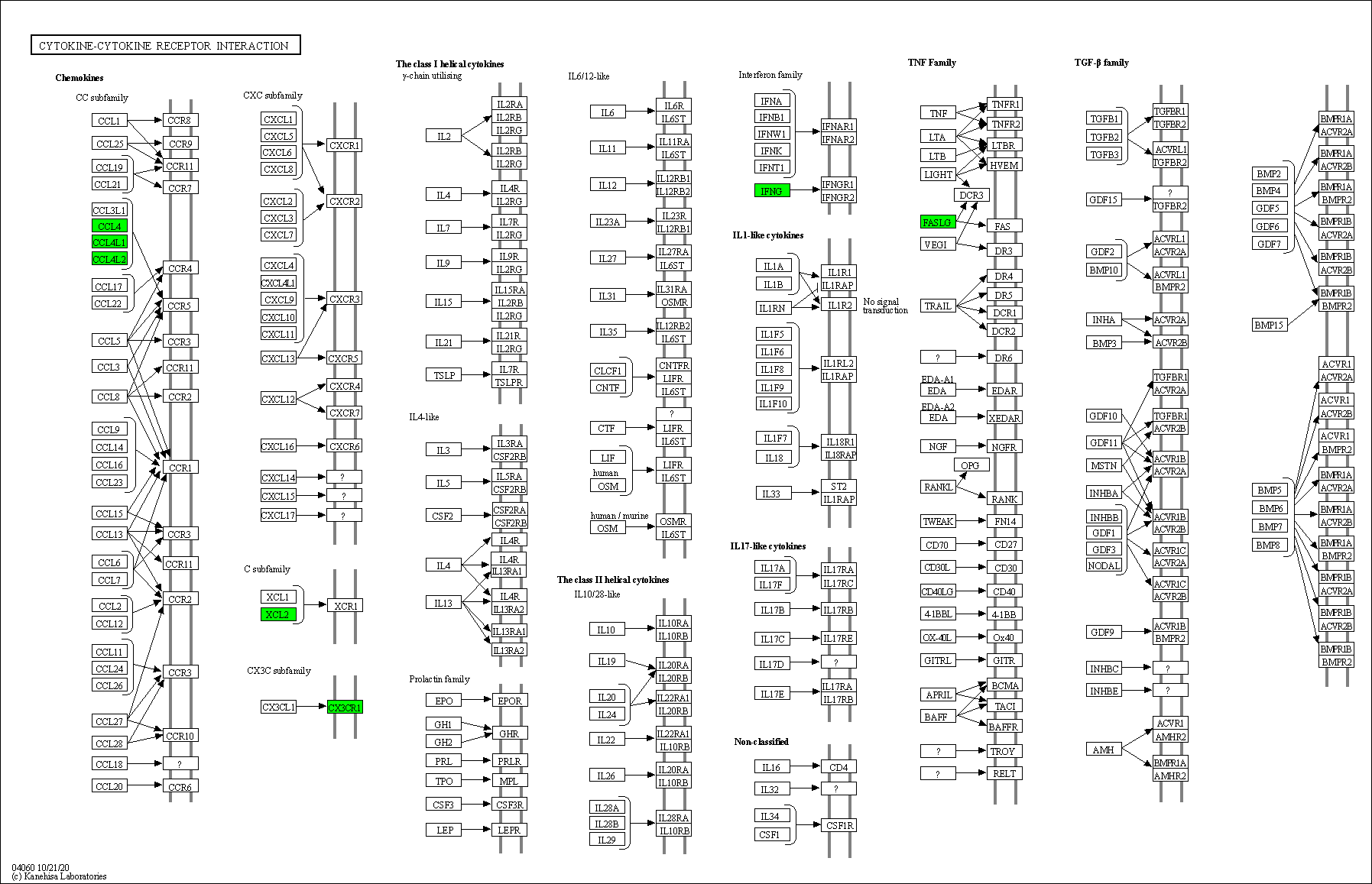


**Supplementary Figure 4:**

**
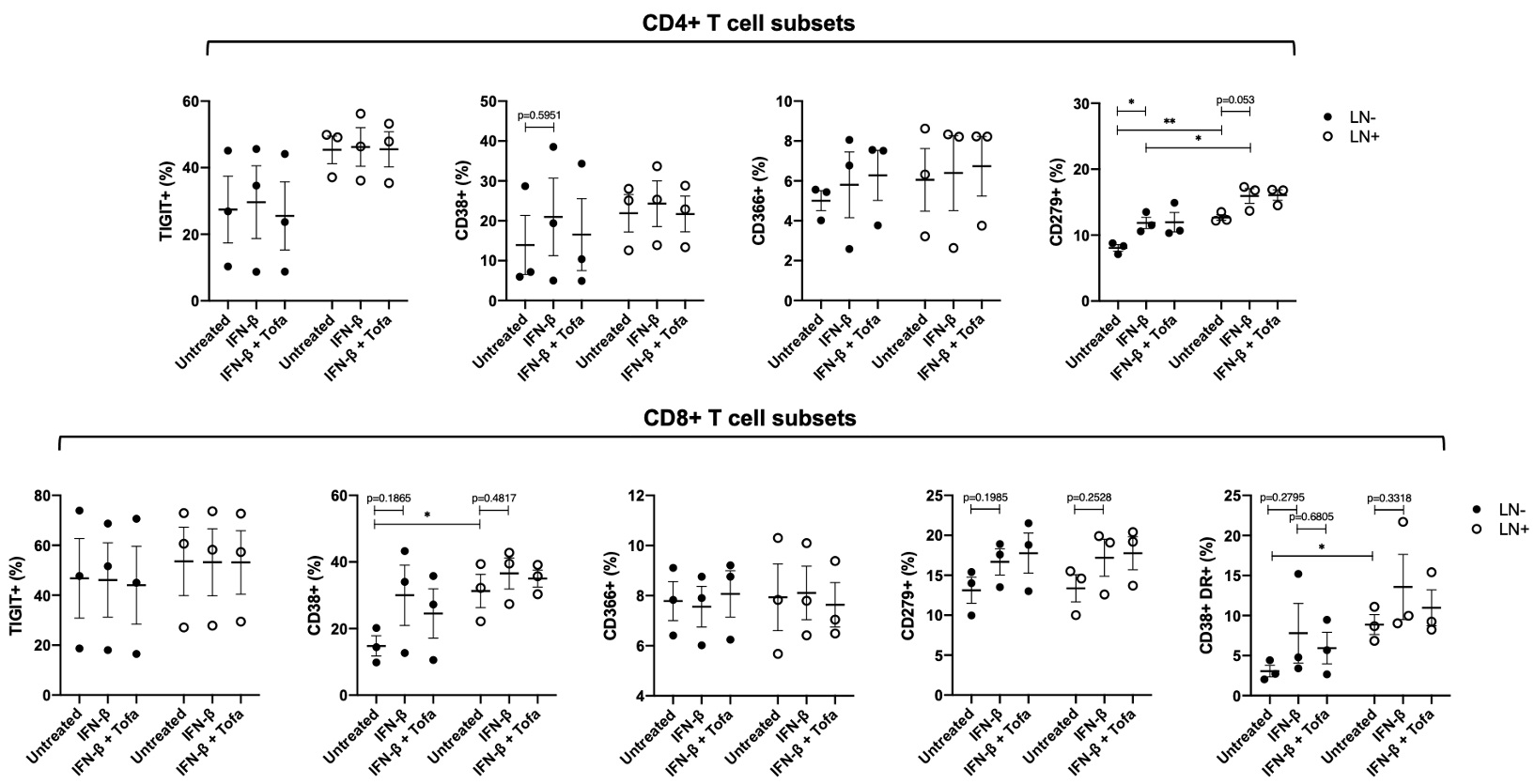
**

Supplementary Table 1: The clinical characteristics of SLE patients & normal controls

| Clinical parameters | SLE(N=91) | Normal(N=27) |
| --- | --- | --- |
| Age (year) | 49.9±11.7 | 36.3±5.7 |
| Male/female | 9/82 | 8/19 |
| Nephritis (+/-) | 23/68 |  |
| SLEDAI (≧6/<6) | 25/66 |  |
| C3 (≧70/<70 mg/dl) | 69/22 |  |
| C4 (≧10/<10 mg/dl) | 74/17 |  |
| Anti-dsDNA Ab (≧130/<130 WHO units) | 35/55 |  |
| Creatine (mean) | 1.00±0.97 |  |
| <1.0 mg/dl | 76 |  |
| 1.0-5 mg/dl | 14 |  |
| >5 mg/dl | 1 |  |
| Anti-RNP antibody (+ >120 AU/ml)  (+/-) | 28/52 |  |
| Anti-SM antibody (+ >120 AU/ml)  (+/-) | 16/64 |  |
| Anti-SSA antibody (+ >120 AU/ml)  (+/-) | 51/31 |  |
| Anti-SSB antibody (+ >120 AU/ml)  (+/-) | 15/67 |  |
| Anti-ACA IgG (+ >20 CU)  (+/-) | 10/67 |  |
| Anti-ACA IgM (+ >20 CU)  (+/-) | 4/61 |  |
| Prednisolone (mg) |  |  |
| ≦10 | 57 |  |
| 10-20 | 23 |  |
| >20 | 11 |  |
| Azathioprine (mg) |  |  |
| ≦50 | 13 |  |
| >50 | 4 |  |
| Mycophenolate (mg) |  |  |
| <1000 | 5 |  |
| ≧1000 | 8 |  |
| HCQ (mg) |  |  |
| ≦200 | 35 |  |
| >200 | 23 |  |

**Supplementary Table 2: The comparison one Co-IRs expression on T cells between SLE nephritis negative and positive**

| **Parameters/markers** | **Mean±SD (Number)** | | **P Value** |
| --- | --- | --- | --- |
| **Nephritis** | **Negative** | **Positive** |  |
| CD4+CD279+ | 6.162 ± 0.6218 (65) | 10.57 ± 1.938 (23) | 0.0056 |
| CD4+TIM3+ | 3.279 ± 0.4234 (66) | 7.378 ± 1.095 (23) | <0.0001 |
| CD4+CTLA4+ | 0.1545 ± 0.04463 (66) | 0.5565 ± 0.1915 (23) | 0.0038 |
| CD4+LAG3+ | 0.9456 ± 0.2004 (68) | 0.8818 ± 0.1656 (22) | 0.862 |
| CD4+CD127+ | 28.55 ± 2.72 (67) | 49.3 ± 2.767 (23) | <0.0001 |
| CD4+TIGIT+ | 24.13 ± 1.297 (67) | 30.14 ± 1.978 (23) | 0.0184 |
| CD8+CD279+ | 7.075 ± 0.9475 (68) | 9.617 ± 1.962 (23) | 0.204 |
| CD8+TIM3+ | 10.59 ± 1.041 (68) | 18.38 ± 1.858 (23) | 0.0003 |
| CD8+CTLA4+ | 0.1538 ± 0.03231 (65) | 0.313 ± 0.04415 (23) | 0.0099 |
| CD8+LAG3+ | 0.2877 ± 0.06649 (65) | 0.3818 ± 0.07719 (22) | 0.4463 |
| CD8+CD127+ | 19.94 ± 2.074 (67) | 29.82 ± 2.773 (23) | 0.013 |
| CD8+TIGIT+ | 31.86 ± 1.76 (67) | 44.38 ± 3.271 (23) | 0.0007 |
| CD8+CD160+ | 31.2 ± 1.77 (66) | 40.82 ± 2.944 (23) | 0.0068 |
| CD8+CD244+ | 1.025 ± 0.2473 (65) | 2.523 ± 0.7825 (22) | 0.0178 |
| CD8+CD38+ | 24.65 ± 2.152 (28) | 27.47 ± 3.506 (23) | 0.4797 |
| CD3+CD160+ | 13.88 ± 0.9825 (67) | 24.21 ± 2.93 (23) | <0.0001 |
| CD3+CD244+ | 3.554 ± 0.9153 (67) | 8.55 ± 1.912 (22) | 0.0115 |
| CD3+CD279+ | 5.314 ± 0.5082 (65) | 8.535 ± 1.609 (23) | 0.0135 |
| CD3+TIGIT+ | 30.08 ± 1.753 (68) | 37.93 ± 3.73 (23) | 0.0372 |

**Supplementary Table 3: The comparison one Co-IRs expression on T cells between SLE SLEDAI <6 and ≧6**

| **Parameters/markers** |  | **Mean±SD (Number)** | | **P Value** |
| --- | --- | --- | --- | --- |
| **SLEDAI Score** |  | **<6** | **≧6** |  |
| CD4+CD279+ |  | 6.338 ± 0.6502 (64) | 8.591 ± 1.731 (22) | 0.1364 |
| CD4+TIM3+ |  | 3.659 ± 0.4579 (63) | 4.659 ± 0.9073 (22) | 0.291 |
| CD4+CTLA4+ |  | 0.1323 ± 0.02599 (62) | 0.2636 ± 0.08986 (22) | 0.0594 |
| CD4+LAG3+ |  | 0.7222 ± 0.1211 (63) | 0.6182 ± 0.146 (22) | 0.6417 |
| CD4+CD127+ |  | 31.01 ± 2.816 (64) | 40.04 ± 4.118 (23) | 0.0928 |
| CD4+TIGIT+ |  | 24.5 ± 1.275 (64) | 28.07 ± 2.38 (23) | 0.1674 |
| CD8+CD279+ |  | 6.815 ± 0.9022 (65) | 7.791 ± 1.725 (23) | 0.5952 |
| CD8+TIM3+ |  | 11.58 ± 1.129 (65) | 13.41 ± 1.805 (23) | 0.4035 |
| CD8+CTLA4+ |  | 0.171 ± 0.03336 (62) | 0.213 ± 0.04185 (23) | 0.4886 |
| CD8+LAG3+ |  | 0.3097 ± 0.06902 (62) | 0.2636 ± 0.07546 (22) | 0.7125 |
| CD8+CD127+ |  | 21.36 ± 2.2 (64) | 24.29 ± 2.882 (23) | 0.4728 |
| CD8+TIGIT+ |  | 32.8 ± 1.744 (64) | 39.44 ± 3.843 (23) | 0.0769 |
| CD8+CD160+ |  | 31.95 ± 1.743 (64) | 37.41 ± 3.599 (22) | 0.1373 |
| CD8+CD244+ |  | 0.9571 ± 0.1999 (63) | 1.005 ± 0.2377 (20) | 0.9001 |
| CD8+CD38+ |  | 23.58 ± 1.937 (31) | 26.19 ± 4.017 (17) | 0.5114 |
| CD3+CD160+ |  | 14.32 ± 1.012 (64) | 22.22 ± 3.09 (23) | 0.0022 |
| CD3+CD244+ |  | 4.105 ± 0.9621 (65) | 6.23 ± 1.86 (23) | 0.2806 |
| CD3+CD279+ |  | 5.556 ± 0.5542 (64) | 7.735 ± 1.666 (23) | 0.1117 |
| CD3+TIGIT+ |  | 29.72 ± 1.693 (65) | 34.75 ± 3.576 (23) | 0.1598 |

**Supplementary Table 4: The comparison one Co-IRs expression on T cells between SLE C3 ≧70 and <70**

| **Parameters/markers** | **Mean±SD (Number)** | | **P Value** |
| --- | --- | --- | --- |
| **C3** | **≧70** | **<70** |  |
| CD4+CD279+ | 7.109 ± 0.8234 (69) | 10.91 ± 1.973 (22) | 0.0408 |
| CD4+TIM3+ | 4.138 ± 0.4901 (68) | 3.542 ± 0.5771 (19) | 0.5439 |
| CD4+CTLA4+ | 0.1567 ± 0.02863 (67) | 0.28 ± 0.09884 (20) | 0.1033 |
| CD4+LAG3+ | 0.9623 ± 0.1975 (69) | 0.8238 ± 0.1713 (21) | 0.71 |
| CD4+CD127+ | 32.48 ± 2.658 (68) | 38.11 ± 4.934 (22) | 0.3042 |
| CD4+TIGIT+ | 24.94 ± 1.3 (68) | 27.92 ± 2.178 (22) | 0.2549 |
| CD8+CD279+ | 7.003 ± 0.9539 (69) | 9.959 ± 1.937 (22) | 0.1451 |
| CD8+TIM3+ | 12.1 ± 1.072 (69) | 14 ± 2.217 (22) | 0.4056 |
| CD8+CTLA4+ | 0.191 ± 0.03116 (67) | 0.2095 ± 0.05892 (21) | 0.7758 |
| CD8+LAG3+ | 0.2485 ± 0.04848 (66) | 0.39 ± 0.1041 (20) | 0.1812 |
| CD8+CD127+ | 20.8 ± 2.013 (68) | 27.6 ± 3.409 (22) | 0.0956 |
| CD8+TIGIT+ | 35.24 ± 1.907 (68) | 34.5 ± 3.362 (22) | 0.8485 |
| CD8+CD160+ | 34.95 ± 1.852 (68) | 29.59 ± 2.812 (21) | 0.149 |
| CD8+CD244+ | 1.518 ± 0.3494 (67) | 1.02 ± 0.2832 (20) | 0.4527 |
| CD8+CD38+ | 24.12 ± 2.184 (38) | 31.19 ± 4.105 (13) | 0.117 |
| CD3+CD160+ | 17.42 ± 1.474 (69) | 15.38 ± 1.809 (22) | 0.4693 |
| CD3+CD244+ | 4.971 ± 1.096 (68) | 5.818 ± 1.822 (22) | 0.6995 |
| CD3+CD279+ | 5.245 ± 0.5207 (66) | 9.191 ± 1.728 (22) | 0.0041 |
| CD3+TIGIT+ | 30.94 ± 2.035 (69) | 35.57 ± 2.237 (22) | 0.2295 |

**Supplementary Table 5: The comparison one Co-IRs expression on T cells between SLE C4 ≧10 and <10**

| **Parameters/markers** | **Mean±SD (Number)** | | **P Value** |
| --- | --- | --- | --- |
| **C4** | **≧10** | **<10** |  |
| CD4+CD279+ | 7.605 ± 0.8903 (74) | 9.865 ± 1.786 (17) | 0.2724 |
| CD4+TIM3+ | 4.199 ± 0.4728 (72) | 3.093 ± 0.5131 (15) | 0.3026 |
| CD4+CTLA4+ | 0.1681 ± 0.03076 (72) | 0.4125 ± 0.1793 (16) | 0.023 |
| CD4+LAG3+ | 0.6569 ± 0.09346 (72) | 0.9333 ± 0.2267 (15) | 0.2313 |
| CD4+CD127+ | 33.33 ± 2.686 (73) | 36.12 ± 4.673 (17) | 0.6436 |
| CD4+TIGIT+ | 25.27 ± 1.25 (73) | 27.36 ± 2.543 (17) | 0.4673 |
| CD8+CD279+ | 7.536 ± 0.9827 (74) | 8.506 ± 1.838 (17) | 0.6653 |
| CD8+TIM3+ | 12.92 ± 1.121 (74) | 10.95 ± 1.796 (17) | 0.4319 |
| CD8+CTLA4+ | 0.1944 ± 0.03152 (71) | 0.2 ± 0.05491 (17) | 0.9359 |
| CD8+LAG3+ | 0.23 ± 0.04366 (70) | 0.5063 ± 0.134 (16) | 0.0149 |
| CD8+CD127+ | 21.94 ± 1.971 (73) | 24.73 ± 3.864 (17) | 0.5356 |
| CD8+TIGIT+ | 34.82 ± 1.815 (73) | 36.09 ± 4.053 (17) | 0.7649 |
| CD8+CD160+ | 34.13 ± 1.789 (72) | 31.84 ± 3.311 (17) | 0.5714 |
| CD8+CD244+ | 1.508 ± 0.333 (71) | 0.9375 ± 0.2808 (16) | 0.4278 |
| CD8+CD38+ | 24.31 ± 2.128 (41) | 32.51 ± 4.539 (10) | 0.0976 |
| CD3+CD160+ | 15.19 ± 1.059 (71) | 17.46 ± 2.425 (17) | 0.3603 |
| CD3+CD244+ | 5.24 ± 1.078 (73) | 3.531 ± 1.286 (16) | 0.4759 |
| CD3+CD279+ | 6.758 ± 0.7575 (74) | 5.938 ± 1.082 (16) | 0.6323 |
| CD3+TIGIT+ | 32.49 ± 1.829 (74) | 30.22 ± 3.822 (17) | 0.5934 |
| CD3+NKG2C+ | 8.929 ± 1.467 (73) | 6.1 ± 1.683 (17) | 0.3723 |
| CD56+CD244+ | 5.236 ± 0.962 (73) | 2.967 ± 0.7611 (15) | 0.2961 |
| CD56+CD279+ | 1.428 ± 0.2278 (72) | 2.025 ± 0.8178 (16) | 0.3342 |
| CD56+TIGIT+ | 32.36 ± 2.688 (74) | 26.31 ± 4.483 (17) | 0.3172 |
| CD56+CD57+ | 41.01 ± 3.098 (74) | 32.61 ± 5.64 (17) | 0.2337 |
| CD56+NKG2C+ | 19.05 ± 2.444 (74) | 10.44 ± 2.957 (17) | 0.1082 |

**Supplementary Table 6: The comparison one Co-IRs expression on T cells between SLE dsDNA <130 and ≧130**

| **Parameters/markers** | **Mean±SD (Number)** | | **P Value** |
| --- | --- | --- | --- |
| **dsDNA** | **<130** | **≧130** |  |
| CD4+CD279+ | 5.516 ± 0.6812 (49) | 9.756 ± 1.44 (32) | 0.0041 |
| CD4+TIM3+ | 3.894 ± 0.5916 (50) | 3.823 ± 0.5966 (31) | 0.936 |
| CD4+CTLA4+ | 0.12 ± 0.029 (50) | 0.22 ± 0.05722 (30) | 0.0883 |
| CD4+LAG3+ | 0.568 ± 0.09741 (50) | 0.5931 ± 0.1147 (29) | 0.8717 |
| CD4+CD127+ | 30.95 ± 3.106 (51) | 32.25 ± 3.739 (31) | 0.7939 |
| CD4+TIGIT+ | 24.28 ± 1.512 (51) | 26.31 ± 1.825 (31) | 0.4015 |
| CD8+CD279+ | 6.265 ± 1.034 (51) | 9.272 ± 1.454 (32) | 0.0877 |
| CD8+TIM3+ | 12.2 ± 1.174 (51) | 13.48 ± 1.991 (32) | 0.5537 |
| CD8+CTLA4+ | 0.1388 ± 0.0282 (49) | 0.1935 ± 0.04274 (31) | 0.2683 |
| CD8+LAG3+ | 0.302 ± 0.07664 (50) | 0.2586 ± 0.08238 (29) | 0.7157 |
| CD8+CD127+ | 21.37 ± 2.522 (51) | 20.46 ± 2.424 (31) | 0.8091 |
| CD8+TIGIT+ | 32.68 ± 2.073 (51) | 35.31 ± 2.911 (31) | 0.4543 |
| CD8+CD160+ | 32.42 ± 2.045 (49) | 32.63 ± 2.698 (32) | 0.9503 |
| CD8+CD244+ | 0.9396 ± 0.219 (48) | 0.7276 ± 0.1991 (29) | 0.512 |
| CD8+CD38+ | 23.94 ± 2.143 (26) | 29.97 ± 4.359 (17) | 0.1777 |
| CD3+CD160+ | 14.23 ± 1.213 (50) | 17.84 ± 2.016 (32) | 0.1071 |
| CD3+CD244+ | 3.124 ± 0.7853 (49) | 1.382 ± 0.3604 (28) | 0.1097 |
| CD3+CD279+ | 5.188 ± 0.6182 (49) | 6.627 ± 0.9323 (30) | 0.1846 |
| CD3+TIGIT+ | 29.26 ± 1.917 (51) | 30.99 ± 2.694 (32) | 0.5935 |
| CD3+NKG2C+ | 6.922 ± 1.521 (51) | 5.697 ± 1.329 (31) | 0.5811 |
| CD56+CD244+ | 3.814 ± 0.8001 (49) | 2.445 ± 0.5999 (29) | 0.2332 |
| CD56+CD279+ | 1.354 ± 0.2569 (50) | 0.9433 ± 0.3054 (30) | 0.3166 |
| CD56+TIGIT+ | 26.04 ± 2.925 (50) | 34.25 ± 3.664 (32) | 0.0838 |
| CD56+CD57+ | 41.33 ± 3.66 (51) | 34.62 ± 4.57 (32) | 0.2567 |
| CD56+NKG2C+ | 15.59 ± 2.007 (50) | 9.453 ± 1.982 (30) | 0.0453 |

**Supplementary Table 7: The comparison one Co-IRs expression on T cells between SLE before and following therapy**

| No. | Age/sex | Nephritis/Interval | Proteinuria | SLEDAI  Score | C3 | C4 | dsDNA |
| --- | --- | --- | --- | --- | --- | --- | --- |
| 1 | 58/F | 1/1 (10.5Ms) | 100/100 | 8/6 | 83.2/92.4 | 11.1/15.5 | 408.8/330.9 |
| 2 | 59/F | 1/0 (12Ms) | 100/- | 8/2 | 83.2/87.7 | 22/22.1 | 312.9/188 |
| 3 | 57/F | 1/0 (13Ms) | 1000/- | 6/2 | 89.4/54.8 | 11.5/4.9 | <40.5/65.8 |
| 4 | 47/M | 1/1 (11Ms) | 100/100 | 4/2 | 81.6/94.6 | 22.8/22.9 | <40.5/<40.5 |
| 5 | 42/F | 1/1 (15Ms) | 1000/300 | 6/4 | 45.5/83.4 | 11.8/17.4 | 108.3/139.6 |
| 6 | 43/F | 0/0 (12Ms) | -/- | 2/2 | 87.1/69.4 | 16.1/6.5 | <40.5/<40.5 |
| 7 | 64/F | 0/0 (13Ms) | -/- | 2/2 | 66/79.5 | 11.5/12.2 | <40.5/<40.5 |
| 8 | 45/F | 0/0 (23Ms) | -/- | 0/2 | 104/65.8 | 20/11.2 | 67/104.2 |
| 9 | 41/F | 1/1 (7Ms) | 300/300 (ESRD) | 6/4 | 65.9/105 | 25.3/31.7 | 51.8/<40.5 |
| 10 | 57/F | 0/1 (10Ms) | -/1000 | 4/6 | 79.4/77 | 8.6/9.62 | 209/85.3 |
| 11 | 27/F | 1/1 (21Ms) | 300/300 | 6/11 | 76/66.5 | 14.7/9.32 | 429.6/306.3 |
| 12 | 27/F | 1/0 (14Ms) | 300/- | 10/2 | 35/77.8 | 3.15/5.07 | 318.5/142.2 |
| 13 | 63/F | 1/0 (9Ms) | 1000/100 | 12/3 | 33.4/109 | 4.33/35.2 | 331.5/<40.5 |
| 14 | 52/F | 1/1 (11Ms) | 300/+ | 4/4 | 112/117 | 24.7/23.8 | 42.8/<40.5 |

| No | CD4/CD279 % | CD4/TIM3 % | CD8/CD279 % | CD8/CD279 MFI | CD8/TIM3 % | CD8/TIM3 MFI | CD4/CTLA4 % | CD8/CTLA4 % | CD8/LAG3 % |
| --- | --- | --- | --- | --- | --- | --- | --- | --- | --- |
| 1 | 5.5/4 | 2.7/1.4 | 5.7/2.8 | 259/381 | 13.5/4.8 | 428/469 | 0/0 | - | - |
| 2 | 8.7/5 | 2.8/1.4 | 9.3/2.1 | 254/406 | 20.1/3.6 | 386/479 | 0/0.1 | 0.4/0 | 0/0 |
| 3 | 0.1/3.7 | 1.7/0.9 | 0.3/4.2 | 203/419 | 13.5/7.2 | 405/467 | 0.1/0 | 0.1/0.1 | 0/0 |
| 4 | 1.1/2.5 | 5.9/2.2 | 2.4/4.2 | 274/426 | 20.5/6.6 | 438/447 | - | 0/0 | - |
| 5 | 5.1/9.4 | 1/1.4 | 4.3/11.4 | 275/432 | 9.2/4.3 | 334/445 | 0/0 | 0.1/0 | 0.3/0 |
| 6 | 2.7/6.1 | 6.2/3.4 | 1.4/1.3 | 261/422 | 8.9/7.4 | 367/502 | 0/0.1 | 0.2/0 | 0.2/0 |
| 7 | 4/4.8 | 8.7/1.1 | 4.1/3.5 | 236/410 | 12.2/2.6 | 377/441 | 0.2/0 | 0.5/0 | 1.3/0 |
| 8 | 19.1/5.5 | 6.3/1.2 | 26.8/7.5 | 324/424 | 12.1/1.3 | 386/415 | 1.2/0 | 2.6/0 | 0.4/0.1 |
| 9 | 19.9/1.1 | 8.1/1.1 | 9.9/0.5 | 269/371 | 12.8/7 | 395/468 | 0.1/0 | 0.2/0 | 0.8/0 |
| 10 | 8.9/2.3 | 3.1/1.5 | 11.6/2.6 | 297/393 | 10.8/4.4 | 375/4.4 | 0.1/0 | 0.3/0 | 0.2/0 |
| 11 | 30.1/10.4 | 11.1/6.5 | 24.5/11.2 | 313/432 | 29/20 | 489/536 | 1/0 | 0.7/0 | 1/0 |
| 12 | 6.9/9.3 | 22.4/1.3 | 6.3/8 | 244/396 | 11.2/3.7 | 414/446 | 1.7/0 | 0.6/0 | 0.9/0.1 |
| 13 | 21.8/6.5 | 13.4/8.3 | 19.6/4.1 | - | 32.9/19.8 | - | 0.2/0 | 0/0 | 0.3/0.2 |
| 14 | 15/3.5 | 8.4/1.8 | 8.9/1.3 | 299/405 | 24/5.8 | 392/426 | 0.1/0 | 0.2/0 | 0.1/0 |

| No | CD4/CD127 % | CD8/CD127 % | CD8/TIGIT % | CD8/CD160 % | CD8/CD160 MFI | CD8/CD244 % | CD8/CD244 MFI | CD3/CD160 % | CD3/CD244 % |
| --- | --- | --- | --- | --- | --- | --- | --- | --- | --- |
| 1 | 41.1/9.2 | 25.7/9.6 | 45.2/32.6 | 26.8/25.6 | 636/629 | 1.4/0.1 | 274/426 | 9.7/11.4 | 5.3/0.2 |
| 2 | 41/11 | 31.9/3.6 | 39.6/41.6 | 34.2/38.3 | 576/728 | 0.9/0.1 | 238/480 | 14.7/21.3 | 3/0.2 |
| 3 | 61.2/7.3 | 24.9/3.6 | 49.8/36.1 | 48.5/31.6 | 657/813 | 0.3/0.8 | 400/390 | 23.3/12.5 | 0.2/2.4 |
| 4 | - | - | - | 33.4/20.7 | 613/798 | 3.5/0.2 | 277/586 | - | - |
| 5 | 47.8/12.8 | 46.2/5.7 | 29.7/38.8 | 17.8/23 | 592/574 | 0.3/0 | - | 6.8/11.2 | 2.6/0.1 |
| 6 | 37.9/9.8 | 12.3/1.5 | 32.9/24.8 | 61.7/46.7 | 829/858 | 2.8/0 | - | 33.2/24.4 | 21.7/1.2 |
| 7 | 62.4/25.9 | 29.3/10.1 | 50.4/37.9 | 36.3/19.5 | 691/642 | 4.2/0 | - | 12.8/6.1 | 25.3/0.1 |
| 8 | 73.7/13.7 | 52.5/9.4 | 30.6/0 | 42.8/31.7 | 740/699 | 0.8/0.2 | 251/662 | 26.4/18.3 | 18.6/0.3 |
| 9 | 46.6/29.8 | 32.1/24.8 | 51.1/27.1 | 25.1/11.2 | 544/685 | 0.8/0 | - | 18/5.6 | 0.7/0.1 |
| 10 | 55.6/19.5 | 40.3/15.1 | 19.2/10.2 | 12.2/6.7 | 541/641 | 0.4/0 | - | 5.7/3.2 | 0.4/0.1 |
| 11 | 54.3/19.3 | 27.4/6.4 | 53.8/55.9 | 59.4/30.4 | 702/735 | 13.8/0.1 | 258/522 | 35.9/18.8 | 39.8/0.1 |
| 12 | 56.1/18.3 | 48.4/17.1 | 51/22.8 | 23.6/22.2 | 550/681 | 1.8/0.2 | 266/581 | 18.3/15.7 | 13.7/1.7 |
| 13 | 46.1/11.3 | 14.9/5.3 | 35.7/16.8 | 51.4/45.6 | - | 0.2/0.4 | - | 21.9/19.2 | 1.9/0.2 |
| 14 | 34.6/4.8 | 32.1/9.2 | 21.2/10.9 | 24.8/15.8 | 685/670 | 0.6/0 | - | 12.8/7.6 | 0.5/0.1 |

| No | CD56/CD160 % | CD56/CD244 % | CD3/CD279 % | CD3/TIGIT % | CD56/TIGIT % | CD3/NKG2C % | CD3/NKG2C MFI |
| --- | --- | --- | --- | --- | --- | --- | --- |
| 1 | 78.9/50.2 | 10.5/0 | 4.2/3.8 | 40.9/39.1 | 58.5/37.1 | 3/0.1 | 252/1077 |
| 2 | 53.2/82.1 | 2/1.1 | 8.1/3 | 46.6/34.9 | 71.5/29.4 | 13.7/0.5 | 353/525 |
| 3 | 63.6/70 | 2.3/5 | 0.2/4.5 | 35.5/3.2 | 33.7/31.6 | 0.1/0.2 | 253/465 |
| 4 | - | - | - | - | - | 21.8/0.7 | 456/390 |
| 5 | 66.4/29.4 | 0/0 | 4.1/6.3 | 31.7/42.9 | 29.5/8.9 | 5.2/0.2 | 267/556 |
| 6 | 89.8/46.6 | 22.3/1.7 | 7.5/3.9 | 44.5/25.7 | 53.2/21.1 | 25.1/0 | - |
| 7 | 88/63 | 17/0 | 3/3.9 | 54.9/30.5 | 46.1/10.2 | 31.9/0.9 | 289/359 |
| 8 | 70.3/56.4 | 7.8/2.6 | 15.5/7.7 | 45.6/27 | 32.5/9.3 | 24.3/3.4 | 296/461 |
| 9 | 58.5/56.5 | 9.4/0 | 11.6/1.7 | 44/30.4 | 53.6/34.9 | 2.4/0.1 | 282/367 |
| 10 | 52.6/40.6 | 2.7/0.4 | 9.2/2.6 | 15.6/12.6 | 37.2/15.8 | 1.3/0.1 | 257/428 |
| 11 | 73.9/32.3 | 42/1 | 20.6/11.4 | 60.3/47.1 | 55.9/26 | 21.3/0 | - |
| 12 | 54.8/30 | 5.4/0.4 | 7.4/8.5 | 55.5/23.9 | 14.8/10.6 | 3.7/0.9 | 300/412 |
| 13 | 67.1/70.6 | 3.5/0.5 | 19.3/1.6 | 31.9/17.1 | 32.8/3.3 | - | - |
| 14 | 52.5/44.4 | 1.8/0 | 16.1/2.3 | 43.1/25.7 | 39.1/14.8 | 0.2/0.2 | 357/728 |
